# Composition–Thermometric Properties Correlations in Homodinuclear Eu^3+^ Luminescent Complexes

**DOI:** 10.1021/acs.inorgchem.0c02611

**Published:** 2020-12-10

**Authors:** Luca Bellucci, Gregorio Bottaro, Luca Labella, Valerio Causin, Fabio Marchetti, Simona Samaritani, Daniela Belli Dell’Amico, Lidia Armelao

**Affiliations:** † CNR ICMATE and INSTM, Dipartimento di Scienze Chimiche, Università di Padova, via Marzolo 1, I-35131 Padova, Italy; ‡ Dipartimento di Chimica e Chimica Industriale and CIRCC, Università di Pisa, via Giuseppe Moruzzi 13, I-56124 Pisa, Italy; § Dipartimento di Scienze Chimiche, Università di Padova, via Marzolo 1, I-35131 Padova, Italy

## Abstract

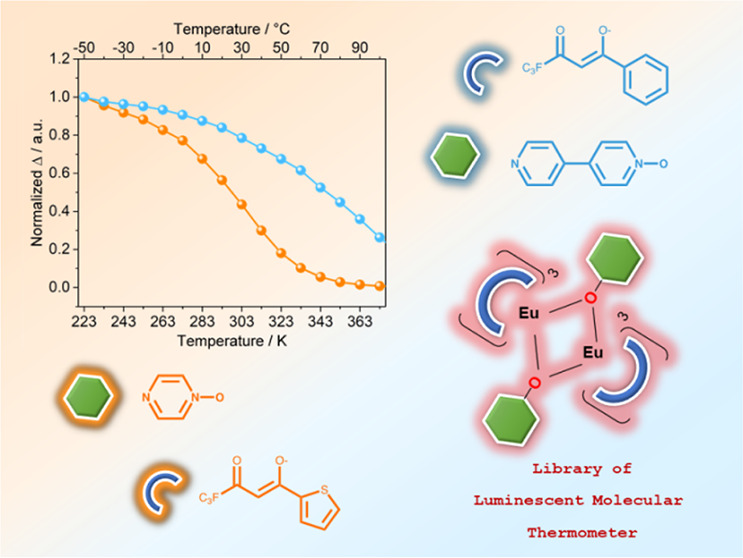

A family of homodinuclear Ln^3+^ (Ln^3+^ = Gd^3+^, Eu^3+^) luminescent complexes with the general formula [Ln_2_(β-diketonato)_6_(*N*-oxide)_*y*_] has been developed to study the effect of the β-diketonato and *N*-oxide ligands on their thermometric properties. The investigated complexes are [Ln_2_(tta)_6_(pyrzMO)_2_] (Ln = Eu (**1**·C_7_H_8_), Gd (**5**)), [Ln_2_(dbm)_6_(pyrzMO)_2_] (Ln = Eu (**2**), Gd (**6**)), [Ln_2_(bta)_6_(pyrzMO)_2_] (Ln = Eu (**3**), Gd (**7**)), [Ln_2_(hfac)_6_(pyrzMO)_3_] (Ln = Eu (**4**), Gd (**8**)) (pyrzMO = pyrazine *N*-oxide, Htta = thenoyltrifluoroacetone, Hdbm = dibenzoylmethane, Hbta = benzoyltrifluoroacetone, Hhfac = hexafluoroacetylacetone, C_7_H_8_ = toluene), and their 4,4′-bipyridine *N*-oxide (bipyMO) analogues. Europium complexes emit a bright red light under UV radiation at room temperature, whose intensity displays a strong temperature (*T*) dependence between 223 and 373 K. This remarkable variation is exploited to develop a series of luminescent thermometers by using the integrated intensity of the ^5^D_0_ → ^7^F_2_ europium transition as the thermometric parameter (Δ). The effect of different β-diketonato and *N*-oxide ligands is investigated with particular regard to the shape of thermometer calibration (Δ vs *T*) and relative thermal sensitivity curves: i.e.. the change in Δ per degree of temperature variation usually indicated as *S*_r_ (% K^–1^). The thermometric properties are determined by the presence of two nonradiative deactivation channels, back energy transfer (BEnT) from Eu^3+^ to the ligand triplet levels and ligand to metal charge transfer (LMCT). In the complexes bearing tta and dbm ligands, whose triplet energy is ca. 20000 cm^–1^, both deactivation channels are active in the same temperature range, and both contribute to determine the thermometric properties. Conversely, with bta and hfac ligands the response of the europium luminescence to temperature variation is ruled by LMCT channels since the high triplet energy (>21400 cm^–1^) makes BEnT ineffective in the investigated temperature range.

## Abstract 2

A family of homodinuclear Eu^3+^ luminescent complexes with the general formula [Ln_2_(β-diketonato)_6_(*N*-oxide)_*y*_] (*y* = 2, 3) was developed to study the effect of the β-diketonato and *N*-oxide ligands on the thermometric properties of the complexes. In this way, an effective tuning of the system’s thermometric properties can be achieved.

## Introduction

During the past decade the increasing use of noncontact techniques for temperature measurements has led to many research efforts in the design of innovative lanthanide-based luminescent thermometers.^1−8^ Lanthanide metal–organic frameworks (LOFs) and coordination polymers (CPs), in particular, due to their thermal stability^2,9−17^ have been extensively explored as luminescent thermometers in a wide temperature range, from ∼600 K down to cryogenic temperatures (<100 K).^12,18^ Moreover, the thermometric properties of these compounds can be tuned through a selective modification of the different building blocks: e.g., metal ions, spacer ligands, and guest molecules.^14^ Less extensive investigations, at variance, have been devoted to the temperature dependence of luminescent properties of discrete lanthanide β-diketonato complexes,^19−26^ though these compounds, being easily processable, are suitable for the development of functional materials for temperature measurements.^27−29^ Europium complexes with a β-diketonato triplet energy of around 20.000–25.000 cm^–1^,^30−33^ are suitable for temperature sensing between 223 and 373 K, an interval that covers the physiological window (298–323 K) and the working range of many integrated circuits.^21,26,34^ The temperature-dependent luminescent properties of these complexes can be easily modulated by changing the lateral substituents on the β-diketonato ligand, as showed in a pioneering work by Sato and Wada.^35^ Although intensity-based ratiometric luminescent thermometers have been the most greatly studied, the development of single-emitter (single-transition) thermometers is still very important for fluidodynamic and aerodynamic applications, where luminescent complexes are used in the so-called temperature-sensitive paints (TSPs) to map the surface temperature distributions. With TSPs the drawbacks deriving from the use of single emitters are bypassed by using the ratio of an image taken under test conditions to an image taken at a known reference condition.^36−38^ Recently, we focused on lanthanide coordination chemistry with the heterotopic divergent *N*-oxide ligand 4,4′-bipyridine *N*-oxide (bipyMO).^39−41^ The different affinities of 4f metal ions toward O- and N-donor ligands afford the synthesis of lanthanide dinuclear complexes with the composition [Ln_2_(β-diketonate)_6_(bipyMO)_*x*_] (*x* = 2, 3 depending on the β-diketonate) where the oxygen atom of bipyMO bridges two lanthanide ions and the nitrogen donor atom is not coordinated.^41−44^ In this context, we developed a family (16 members) of homodinuclear Eu^3+^ and Gd^3+^ luminescent compounds to study the effect of the β-diketonato and *N*-oxide ligands on the thermometric properties of the complexes. We used four β-diketonato ligands with different steric and electronic properties commonly used for the synthesis of europium luminescent derivatives: thenoyltrifluoroacetone (Htta), dibenzoylmethane (Hdbm), benzoyltrifluoroacetone (Hbta), and hexafluoroacetylacetone (Hhfac). As *N*-oxide ancillary ligands we employed both bipyMO and pyrazine *N*-oxide (pyrzMO), the monoaromatic ring analogue of bipyMO, only scarcely investigated in lanthanide coordination chemistry to date.^45−47^ The studied family is composed of [Ln_2_(tta)_6_(pyrzMO)_2_] (Ln = Eu (**1**·C_7_H_8_), Gd (**5**)), [Ln_2_(dbm)_6_(pyrzMO)_2_] (Ln = Eu (**2**), Gd (**6**)), [Ln_2_(bta)_6_(pyrzMO)_2_] (Ln = Eu (**3**), Gd (**7**)), [Ln_2_(hfac)_6_(pyrzMO)_3_] (Ln = Eu (**4**), Gd (**8**)), and their bipyMO-based analogues (**1b**–**8b**).^41^ All of the europium complexes showed an intense red emission at room temperature under UV radiation and a strong temperature (*T*) dependence of europium emission intensity between 223 and 373 K. This remarkable variation is exploited to develop a series of luminescent thermometers by using the integrated intensity of the europium ^5^D_0_ → ^7^F_2_ transition as a thermometric parameter (Δ). The effect of the different β-diketonato and *N*-oxide ligands on the shape of thermometer calibration (Δ vs *T*) curves is investigated and presented in detail.

## Results and Discussion

**Figure 1:**
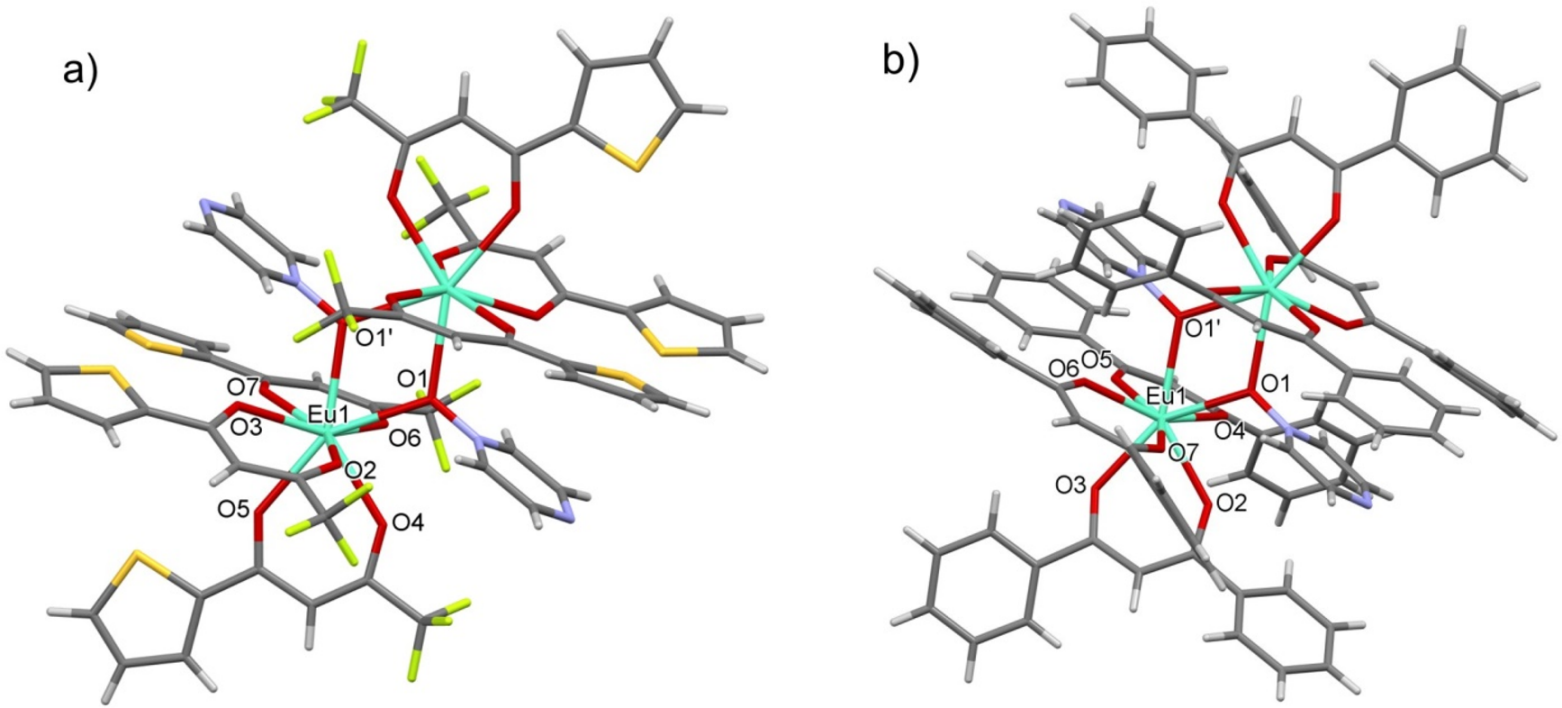
Molecular structures of compounds (a) **1** and (b) **2**.

Since previous studies established the product composition [Ln_2_(β-diketonate)_6_(bipyMO)_2_] (β-diketonate = tta, dbm) regardless of the [Ln(β-diketonate)_3_]/bipyMO molar ratio (1/1 or 2/3),^41^ the reaction between [Eu(β-diketonate)_3_] (β-diketonate = tta, dbm) and pyrzMO was carried out with a 1/1 molar ratio in hot anhydrous toluene. The products [Eu_2_(tta)_6_(pyrzMO)_2_]·C_7_H_8_ (**1**·C_7_H_8_) and [Eu_2_(dbm)_6_(pyrzMO)_2_] (**2**) were obtained in satisfactory yields. Recrystallization of the products in toluene through the diffusion of pentane vapors led to single crystals suitable for X-ray diffraction studies with the composition [Eu_2_(β-diketonate)_6_(pyrzMO)_2_]·*n*C_7_H_8_ (β-diketonate = tta, *n* = 5; β-diketonate = dbm, *n* = 0). The solvated toluene molecules in **1** are partially lost on drying, but a solvent molecule was retained even after a long treatment *in vacuo* at room temperature. The structures of **1** and **2** are characterized by the [Eu_2_(β-diketonate)_6_(pyrzMO)_2_] dinuclear unit, where pyrzMO acts as a bridging ligand only via its oxygen atom (μ-O), leaving the nitrogen site uncoordinated (Figure 1).

In both compounds, europium centers show a coordination number (CN) of 8 and a square-antiprismatic geometry. The different substituents on the β-diketonato ligands slightly influence the shape of europium coordination polyhedra (see Table S2). Complexes **1** and **2** are centrosymmetric with the inversion center placed in the middle of the oxygen atoms of the two pyrzMO ligands.

The observed bridging μ-O is an unprecedented coordination mode for pyrzMO, although it is expected for a bipyMO analogue. The molecular structures of **1** and **2** closely resemble the related bipyMO structures [Ln_2_(β-diketonate)_6_(bipyMO)_2_].^41^

**Figure 2:**
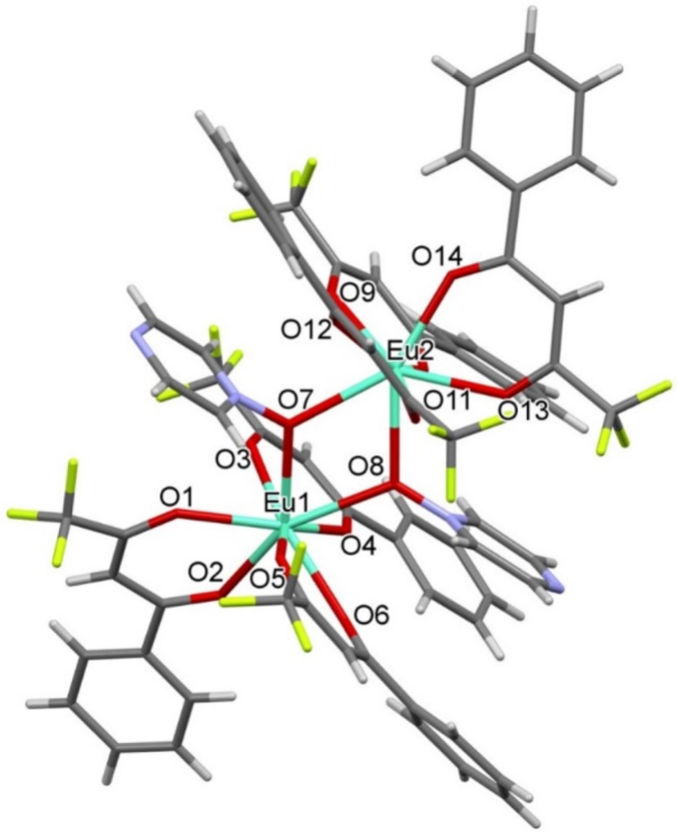
Molecular structure of compound **3**. Only the most populated positions of disordered CF_3_ groups have been represented.

Although the reactivity between [Eu(bta)_3_] and *N*-oxide ligands had not been previously explored, we used a [Eu(bta)_3_]/pyrzMO molar ratio of 1/1, expecting the formation of the [Eu_2_(bta)_6_(pyrzMO)_2_] complex. Indeed, Hbta has steric and electronic properties similar to those of Htta, having a phenyl ring instead of a thienyl group, and a p*K*_a_ value close to that of Htta (p*K*_a_ (Hbta) ≈ 6.1; p*K*_a_(Htta) ≈ 6.3).^48^ Elemental analysis and single-crystal XRD studies confirmed the formation of the expected compound [Eu_2_(bta)_6_(pyrzMO)_2_] (**3**). As in **1** and **2**, the molecular structure presents a europium dinuclear unit with two bridging O-coordinated *N*-oxide hypodentate ligands (Figure 2). Each europium ion has CN = 8 and a square-antiprismatic coordination geometry. However, unlike the case for **1** and **2**, in compound **3** the two europium centers are crystallographically independent and the complex does not have an inversion center. Some geometrical parameters of the coordination polyhedron are given in Table S2. Since no bipyMO analogue had previously been prepared, and the number of bridging *N*-oxide ligands was not assessed, the reaction was repeated using an [Eu(bta)_3_]/pyrzMO molar ratio of 2/3 and compound **3** was still obtained.

For comparison purposes we also prepared the bipyMO analogue with bta ligands: using the same stoichiometric ratio, we obtained [Eu_2_(bta)_6_(bipyMO)_2_] (**3b**; see the Supporting Information)

According to previous studies,^41,43,44^ the reaction between [Eu(hfac)_3_] and a *N*-oxide ligand proceeds with a 2/3 metal/ligand molar ratio. For this reason, [Eu(hfac)_3_] and pyrzMO were reacted using this reaction stoichiometry, yielding the analytically pure compound [Eu_2_(hfac)_6_(pyrzMO)_3_] (**4**).

**Figure 3:**
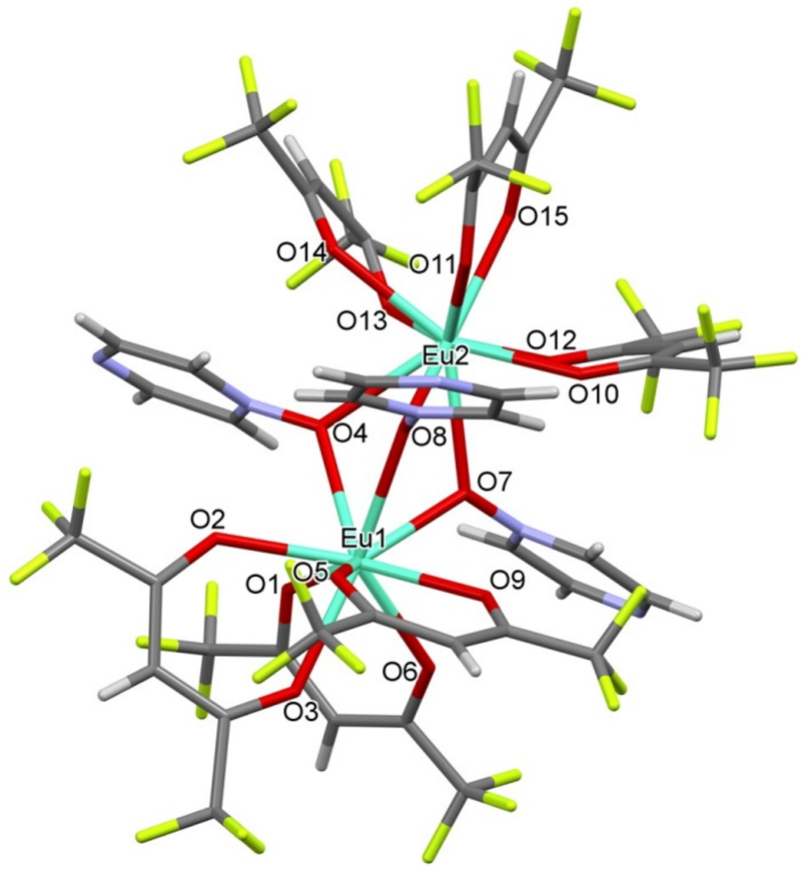
Molecular structure of compound **4**.

By cooling of a toluene solution to −20 °C, single crystals suitable for X-ray diffraction have been recovered. The crystals collapsed, losing solvent, on standing in air. Structural studies showed a [Eu_2_(hfac)_6_(pyrzMO)_3_]·C_7_H_8_ composition, presenting dinuclear molecules with nine-coordinate europium ions and three μ-O-bridging pyrzMO ligands (Figure 3).

Compound **4**·(toluene) has no symmetry elements. Both of the crystallographically independent europium atoms are in a tricapped-trigonal-prismatic coordination, and the two polyhedra share the O4, O7, O8 face. Some geometrical parameters are given in Table S2.

Our results suggest that the tendency of the *N*-oxide ligand to adopt the μ-O bridging coordination mode is independent of the β-diketonato ligand,^41−44^ while the number of *N*-oxide ligands for a dinuclear molecule is ruled by the β-diketonato nature.^41^

Gadolinium analogues of compounds **1**–**4** have been prepared for comparison purposes. With [Gd(β-diketonate)_3_] and pyrzMO in anhydrous toluene as the starting materials, Gd_2_(tta)_6_(pyrzMO)_2_] (**5**), [Gd_2_(dbm)_6_(pyrzMO)_2_] (**6**), [Gd_2_(bta)_6_(pyrzMO)_2_] (**7**), and [Gd_2_(hfac)_6_(pyrzMO)_3_] (**8**) have been obtained in satisfactory yields with IR spectra superimposable with those of the corresponding europium compounds. The related compounds Gd_2_(tta)_6_(bipyMO)_2_] (**5b**), [Gd_2_(dbm)_6_(bipyMO)_2_] (**6b**), [Gd_2_(bta)_6_(bipyMO)_2_] (**7b**), and [Gd_2_(hfac)_6_(bipyMO)_3_] (**8b**) have been similarly obtained using bipyMO as the *N*-oxide ligand (see the Supporting Information).

### Photoluminescence Studies

**Figure 4:**
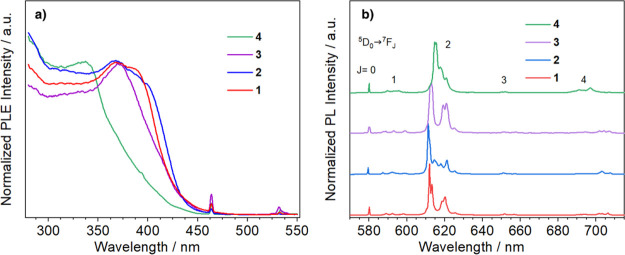
(a) Photoluminescence excitation spectra of compounds **1**–**4**. λ_em_ = 611 nm. (b) Emission spectra of compounds **1**–**4**. λ_exc_ = 350 nm.

All europium complexes emit bright red light upon irradiation across the UV/vis regions, between 350 and 450 nm. Complexes bearing β-diketonato ligands with an increasing number of aromatic rings present a red shift of the excitation maximum from ca. 350 nm in **4** to 380–400 nm in **1**–**3**, as evidenced by the photoluminescence excitation spectra (PLE) (Figure 4a).

Europium emission is achieved thanks to a ligand-mediated sensitization process, the so-called *antenna effect*.^49,50^ The whole process is summarized as follows: (i) light absorption via ligand centered transitions (chromophore) with population of the first excited singlet state, (ii) intersystem crossing from the singlet to the triplet (T) level, and (iii) energy transfer from the triplet level of the chromophore to the lanthanide excited levels, and (iv) metal-centered emission.^51^ The photoluminescence (PL) spectra of compounds **1**–**4** show the typical europium sharp bands in the 570–720 nm range associated with Eu^3+^^5^D_0_ → ^7^F_*J*_ (*J* = 0–4) transitions (Figure 4b).^50,52^ As is usual for europium β-diketonato complexes, all spectra present a strong hypersensitive ^5^D_0_ → ^7^F_2_ transition, which is about 1 order of magnitude more intense than the other ^5^D_0_ → ^7^F_*J*_ (*J* = 0, 1, 3, 4) emission lines.

As in bipyMO analogues,^39−41^ the complexes show different europium coordination numbers (CNs) depending on the number of pyrzMO ligands bonded to the two metal centers: CN = 8 in **1**–**3**; CN = 9 in **4**. The differences in the crystal field components of the ^5^D_0_ → ^7^F_*J*_ multiplets observed in the PL spectra of the complexes are due to small variations in the Eu^3+^ coordination polyhedra related to CNs as well as to R and R′ substituents at the β-diketonato moieties.

The nature of the β-diketonato ligands, and in particular the presence of fluorine atoms, influences the ^5^D_0_ experimental lifetime (τ_obs_), which progressively increases from 0.58 to 0.70 ms (Table 1) in the order dbm ≈ tta < bta < hfac, respectively. Indeed, the introduction of an increasing number of −CF_3_ groups diminishes the probability of a nonradiative deactivation path of the excited state.^53^ Relevant spectroscopic data for pyrzMO derivatives are summarized in Table 1. To study the effects of β-diketonato and *N*-oxide ligands on the PL properties of the complexes, a comparison can be made with the data for bipyMO analogues [Eu_2_(tta)_6_(bipyMO)_2_] (**1b**), [Eu_2_(dbm)_6_(bipyMO)_2_] (**2b**), [Eu_2_(bta)_6_(bipyMO)_2_] (**3b**) (Figure S2), and [Eu_2_(hfac)_6_(bipyMO)_3_] (**4b**), whose room-temperature PL has been previously described.^41^ Useful spectroscopic parameters to consider in this comparison are radiative lifetimes (τ_rad_), intrinsic quantum yield (Φ), absolute photoluminescence quantum yield, and sensitization efficiency (η). Conversely to τ_obs_, the radiative lifetime corresponds to the luminescence lifetime of the ^5^D_0_ level in the absence of nonradiative processes^50^ and it can be calculated from europium emission spectra according to eq 1
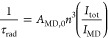
1where *A*_MD,0_ is the spontaneous emission probability *in vacuo* of the ^5^D_0_ → ^7^F_1_ transition (14.65 s^–1^), *I*_tot_ and *I*_MD_ are the integrated areas of the whole emission spectrum and the ^5^D_0_ → ^7^F_1_ transition, respectively, and *n* is the refractive index (*n* ≈ 1.55 at the solid state).^50^ The difference between τ_rad_ and τ_obs_ is attributed to the presence of nonradiative relaxation phenomena that cannot be directly observed. τ_rad_ and τ_obs_ are used to calculate the intrinsic quantum yield (Φ, eq 2.1) and the efficiency (η, eq 2.2) of the sensitization process
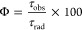
2.1
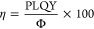
2.2where PLQY is the absolute photoluminescence quantum yield: i.e. an experimental evaluated quantity defined as the ratio between the number of emitted and absorbed photons (PLQY ≤ Φ).

**Table 1:** Experimental Lifetimes (τ_obs_), Radiative Lifetimes (τ_rad_), Photoluminescence Quantum Yields (PLQY), Intrinsic Quantum Yields (Φ), and Sensitization Efficiencies (η) for the Europium Dinuclear Compounds Excited at 350 nm

compound	*T* (cm^–1^)^b^	τ_obs_ (ms)	τ_rad_ (ms)	PLQY (%)	Φ (%)	η (%)
**1** (**1b**)^a^	20200 (20500)	0.59 (0.58)	1.00 (1.02)	35 (55)	59 (57)	59 (96)
**2** (**2b**)^a^	20600 (20500)	0.58 (0.45)	1.17 (0.90)	48 (42)	50 (50)	96 (84)
**3** (**3b**)	21600 (21500)	0.67 (0.60)	1.00 (0.99)	22 (40)	67 (61)	33 (66)
**4** (**4b**)^a^	22100 (22300)	0.70 (0.65)	1.04 (1.02)	10 (30)	67 (64)	15 (47)

a Data from ref (41).

b Experimental values estimated from low-temperature emission spectra of **5**–**8** and **5b**–**8b** (Figure S3) derivatives. A very good agreement with literature values for monomeric β-diketonato complexes (20400,^33^ 20500,^33^ 21450,^32^ and 21900^31^ cm^–1^ for Eu(tta)_3_, Eu(dbm)_3_, Eu(bta)_3_ ,and Eu(hfac)_3_, respectively) is found. The error on the triplet energy values is ±3%.

For all pyrzMO-containing dinuclear complexes except **2**, PLQY values are lower than those of the corresponding bipyMO derivatives and range from 55% for **1b** to 10% for **4**. The sensitization efficiency η shows a similar trend and was nearly 100% for **1b** and **2** and only 15% for **4**. The data in Table 1 also show a correlation between η and the β-diketone triplet energy. In particular, the higher the triplet energy, the greater the difference in the sensitization efficiency between pyrzMO and bipyMO analogues, and the lower their absolute quantum yield. The data in Table 1 show a close relationship between the composition of the complexes and their spectroscopic properties due to the different contributions of nonradiative relaxation of excited states that play a pivotal role in the determination of the thermometric properties of these compounds.

### Thermometric Properties

The luminescence properties of Eu^3+^ β-diketonato complexes having Δ*E*_*T*-Eu_ = *E*(*T*) – *E*(^5^D_0_ → ^7^F_0_) in the 2000–5000 cm^–1^ range are generally more sensitive to temperature variations between 223 and 423 K.^35,54^ On these grounds, we decided to investigate the temperature-dependent europium emission (TDE) of compounds **1**–**4** and **1b**–**4b** in order (i) to study the correlations between the nature of β-diketonato and *N*-oxide ligands with TDE modulation, (ii) to elucidate the observed room-temperature emission, and (iii) to explore the potential of this family of dinuclear complexes as luminescence thermometers.

Prior to investigating the dependence of europium emission from temperature, we performed thermal analysis in air of the microcrystalline powders of the samples. This preliminary step is relevant to check and determine the thermal stability of the complexes in view of their use to study the TDE behavior. The profiles of weight loss for complexes **1**–**4** and **1b**–**4b** (Figure S4) evidenced their stability up to ca. 400 K. In particular, **1** and **1b** present a small step around 380 K that is due to the release of toluene molecules retained in the crystals.

**Figure 5:**
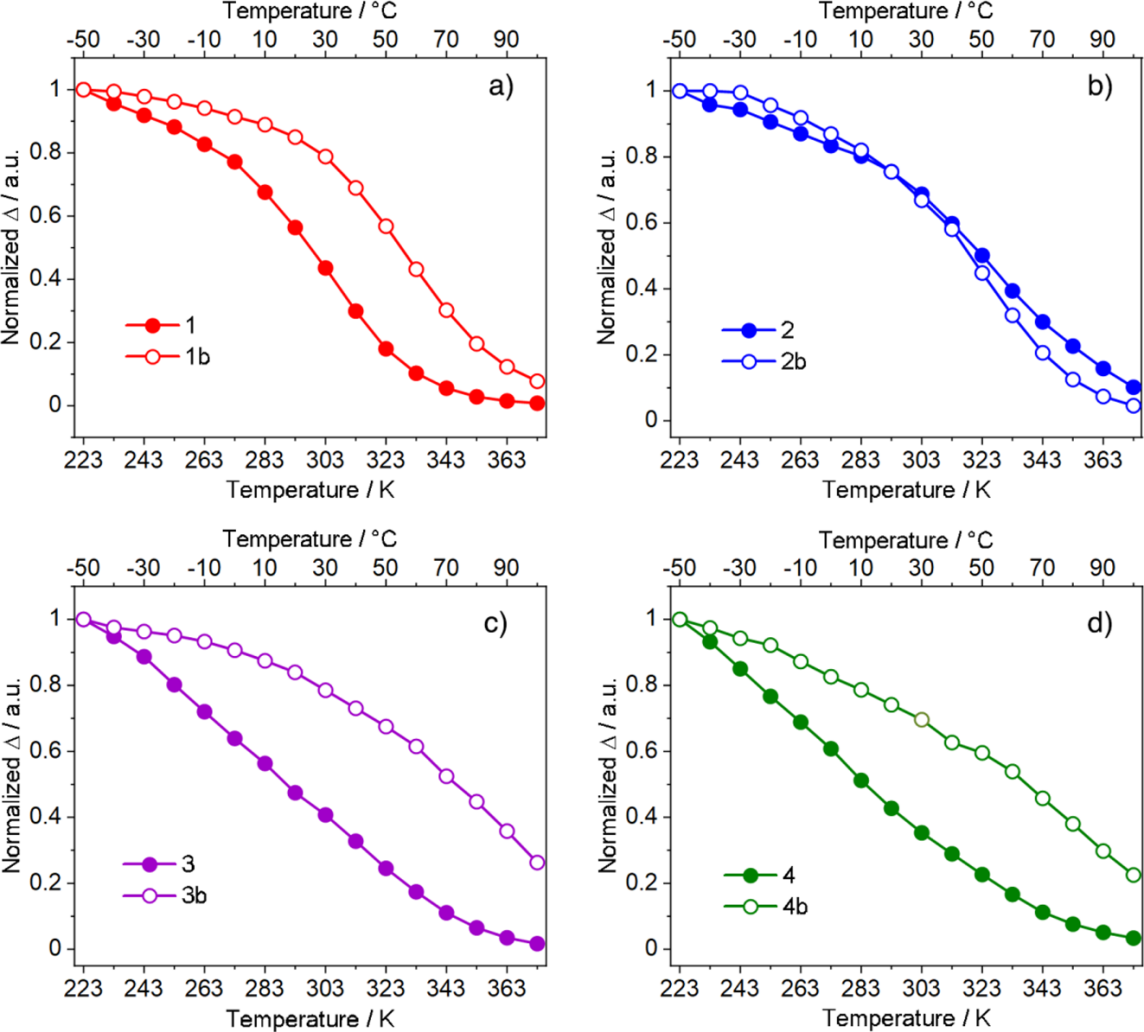
Comparison between the Δ curves of the pyrzMO and the bipyMO derivatives: (a) **1** and **1b**; (b) **2** and **2b**; (c) **3** and **3b**; (d) **4** and **4b**. λ_exc_ = 350 nm.

The intensity of the emitted light decreases as temperature increases in the range 223–373 K (see Figure S5). As a thermometric parameter (Δ) we used the integrated area of the Eu^3+^^5^D_0_ → ^7^F_2_ transition that, in all compounds, makes the main contribution to the total emission (Figure 4b). For a better comparison, Δ vs *T* curves were normalized at 223 K (Figure 5).

Δ curves for complexes **1**–**4** and **1b**–**4b** are a family of sigmoids having slopes sensitive to both β-diketonato and *N*-oxide ligands. Interestingly, for pyrzMO-based compounds the Δ curve variation begins generally at a lower temperature and proceeds more quickly than that of the corresponding bipyMO derivatives. An exception is found for compounds **2** and **2b**, where the Δ curves are almost overlapped. This behavior, together with large differences in the sensitization efficiency for complexes bearing pyrzMO or bipyMO ligands, indicates a correlation between the *N*-oxide moieties and the contribution of nonradiative deactivation pathways to TDE.

**Figure 6:**
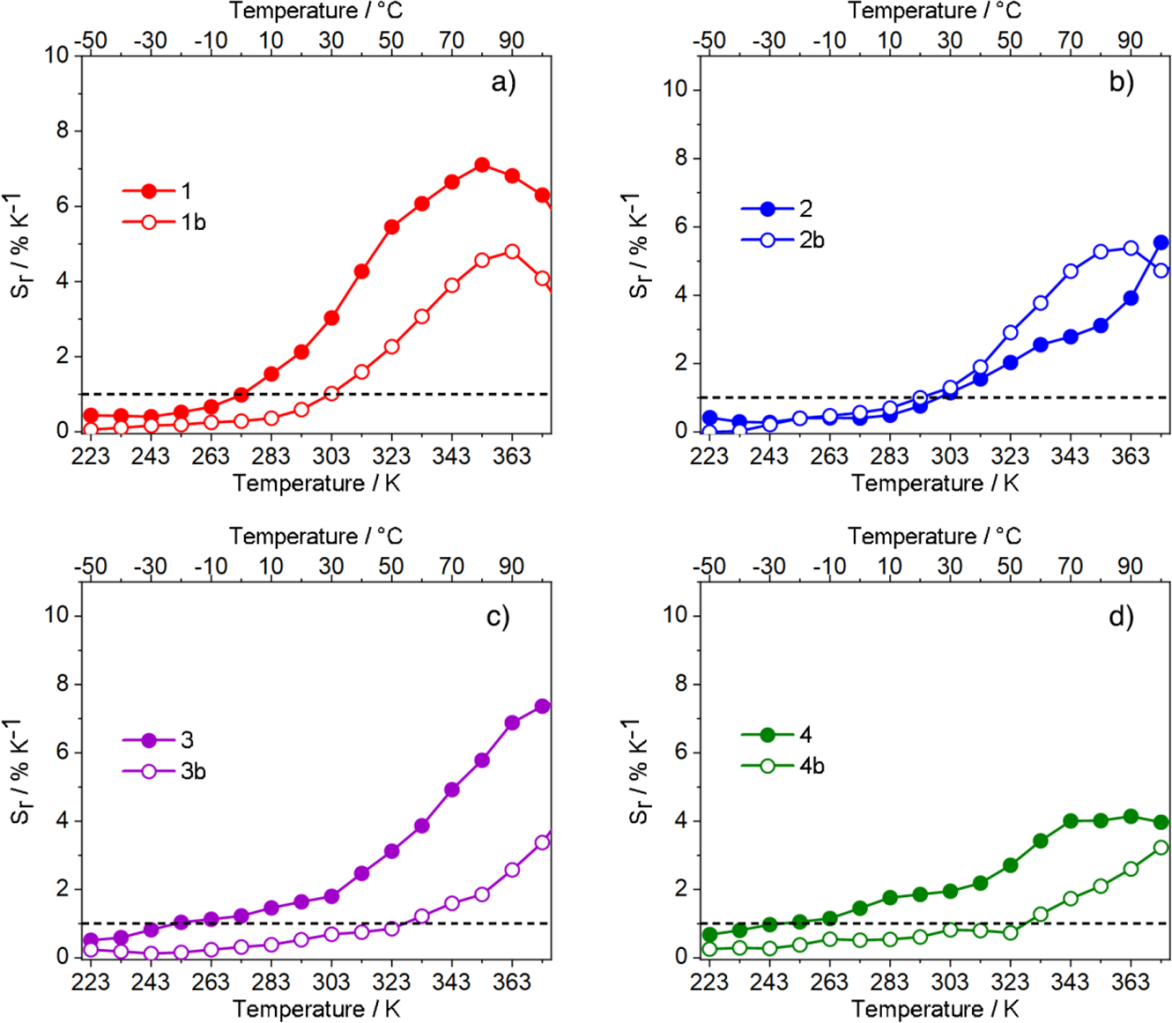
*S*_r_ curves of compounds (a) **1** and **1b**, (b) **2** and **2b**, (c) **3** and **3b**, and (d) **4** and **4b**.

For a better comparison of the thermometric properties, we calculated the relative thermal sensitivity (*S*_r_, expressed in % K^–1^, eq 3): i.e., the figure of merit usually employed for thermometer comparison^55^
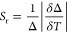
3where Δ is the thermometric parameter and δΔ is the variation of the thermometric parameter per temperature variation (δ*T*). A value of *S*_r_ ≥ 1 is assumed as the quality criterion for using these compounds as highly sensitive luminescent thermometers (Figure 6).^3^

All of the complexes displayed an overall good thermometric response, with *S*_r_ ≥ 1, over *T* ranges from about 40 K up to 130 K. As a general observation, pyrzMO-based complexes show wider applicative temperature intervals (up to 70 K for **4** and **4b**) and higher *S*_r_ values than their corresponding bipyMO analogues. Compound **3**, instead, displayed the highest *S*_r_ maximum of 7.4% K^–1^ at 373 K. These results, summarized in Table 2, show that the design of the complex chemical composition is a practical and effective route to tune the TDE of the systems.

**Table 2:** Applicative Temperature Range, *S*_r_ Maximum Values and Its Corresponding Temperature (*TS*_r_(max)) of Compounds **1**, **1b**, **2**, **2b**, **3**, **3b**, **4**, and **4b**

sample	*TS*_r_ > 1 (K)	applicative range (K)	*S*_r_(max) (% K^–1^)	*TS*_r_(max) (K)
**1**	273	100	7.1	353
**1b**	303	70	4.8	363
**2**	293	80	4.9	373
**2b**	293	80	4.9	343
**3**	253	120	7.4	373
**3b**	333	40	3.4	373
**4**	243	130	4.1	363
**4b**	333	40	3.2	373

The Mott–Seitz (MS) model is commonly used to determine the temperature effect on the quenching of lanthanide luminescence, and it can be used to rationalize the obtained results. The experimental data were fitted to find the number and the activation energies of the main nonradiative deactivation channels^56,57^
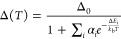
4where Δ_0_ is the value of Δ at 0 K, Δ*E*_*i*_ the activation energy, *α*_*i*_ the ratio between the nonradiative and radiative deactivation probabilities for the considered path, *k*_b_ the Boltzmann constant, and *T* the temperature in K.^58^

As shown in Figure S6, the one-term MS equation is a good model to reproduce our Δ vs *T* experimental curves.

**Figure 7:**
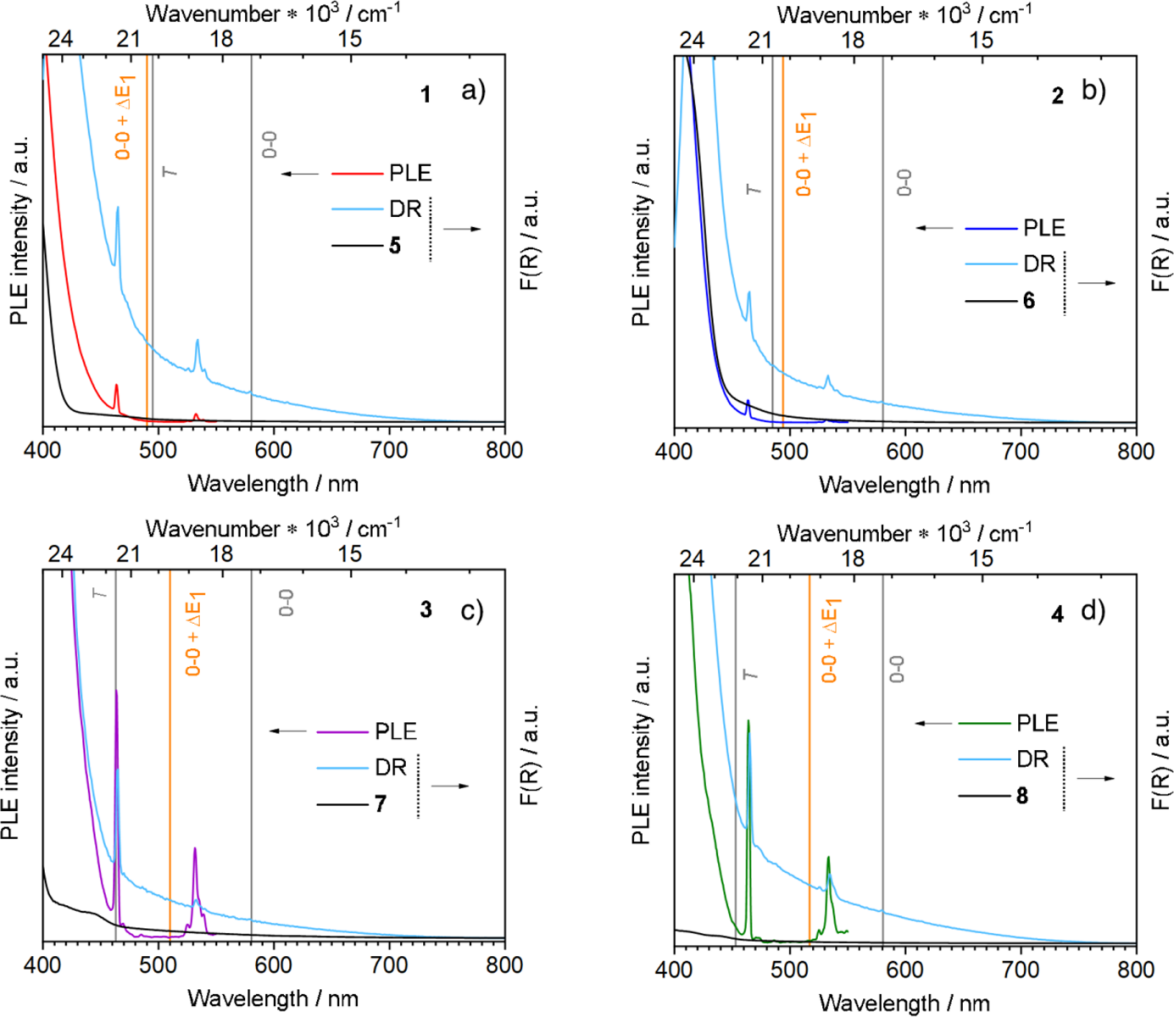
Overlap between the photoluminescence excitation (PLE, left axis) and the absorption (DR, right axis) spectra of (a) **1**, (b) **2**, (c) **3**, and (d) **4**. To better evidence the presence of LMCT transitions, the absorption spectra of Gd complexes **5**–**8** are reported as well. The label “0–0” refers to the energy of the Eu^3+^^5^D_0_ → ^7^F_0_ transition, *T* is the energy of the β-diketonato triplet level, and Δ*E*_1_ is the activation energy determined with the MS equation.

The most common nonradiative deactivation pathways usually considered in the MS model are^59,60^ (i) back energy transfer (BEnT) from the Eu^3+^^5^D_0_ level to the ligand triplet levels, (ii) ligand to metal charge transfer (LMCT), and (iii) multiphonon relaxations. For tta (**1**, **1b**)- and dbm-based (**2**, **2b**) compounds, MS activation energies (Δ*E*_1_, eq 4 and Table 3) are comparable to the Δ*E*_T-Eu_ energy gap, indicating that BEnT is the most active channel in determining the temperature dependence of europium emission. Conversely, the values of Δ*E*_1_ and Δ*E*_T-Eu_ for compounds **3**, **3b**, **4**, and **4b** are quite different, suggesting a different mechanism for the nonradiative depopulation of the excited states. The comparison in the visible region of solid-state absorption spectra (diffuse reflectance) and excitation spectra (Figure 7 and Figure S7) aided in shining some light on this behavior.

**Table 3:** Δ*E*_T-Eu_ and Δ*E*_1_ Energy Values for the Studied Compounds^a^

compound	Δ*E*_T-Eu_ (cm^–1^)	Δ*E*_1_ (cm^–1^)
**1**	2980 ± 606	3109 ± 499
**1b**	3280 ± 615	3409 ± 392
**2**	3380 ± 618	3021 ± 379
**2b**	3330 ± 616	2876 ± 390
**3**	4480 ± 651	2377 ± 350
**3b**	4350 ± 647	2602 ± 286
**4**	4880 ± 663	2116 ± 247
**4b**	5080 ± 669	2109 ± 327

a In the second column is reported the triplet energy level of the β-diketonato ligand.

The absorption spectra of all compounds have extended tails in the visible region up to 700 nm. These signals overlap with ^5^D_0_ → ^7^F_*J*_ manifolds, but they are not able to sensitize Eu^3+^ emission, as evidenced in PLE spectra, where ligand-centered transitions occur below 500 nm. This suggests the existence of not yet considered low-energy levels, able to act as further nonradiative deactivation channels. This situation is often encountered in the presence of low-energy LMCT states (*E*_LMCT_ < 25000 cm^–1^).^50,57,61−63^ A commonly accepted method to reveal such transitions in lanthanoid complexes is a comparison of the absorption spectra of Eu^3+^ and Gd^3+^ compounds. In fact, as the gadolinium first excited state is at ca. 32000 cm^–1^, LMCT transitions in the visible region are not observed. The spectra of Gd complexes **5**–**8** reported in Figure 7 (black curves) do not show bands at λ > 400 nm, thus confirming the presence of LMCT transitions in the absorption spectra of Eu^3+^ complexes. A similar behavior is found for bipyMO-bearing complexes (Figure S7).

The vertical lines in Figures 7 and Figure S7 highlight the energy of (i) the ligand-centered triplet (T), (ii) ^5^D_0_ → ^7^F_0_ transitions labeled as 0–0, and (iii) *E*_MS_ = ^5^D_0_ → ^7^F_0_ + Δ*E*_1_.

In compounds **1**, **1b**, **2**, and **2b** (cf. Figure 7a,b and Figure S7a,b) *E*_MS_ and *T* values are very close, indicating the primary role of back energy transfer in Eu^3+^ luminescence quenching, as already mentioned. Although we cannot exactly determine the energy of LMCT transitions, there is experimental evidence of their contribution from the Δ vs *T* curves (Figure 5). In fact, the observed Δ difference between pyrzMO-bearing complexes (**1** and **2**) and their analogues with bipyMO (**1b** and **2b**) are due to the LMCTs that are more effective in **1** and **2**, as also highlighted by the lower η values for pyrzMO derivatives (Figures 5 and 6 and Table 1).

Instead, in compounds **3**, **3b**, **4**, and **4b** (cf. Figure 7c,d and Figure S7c,d) bearing β-diketonato ligands with high triplet energy, BEnT is less effective and LMCT becomes the quenching channel that rules the thermometric properties.

### Photostability Studies

**Figure 8:**
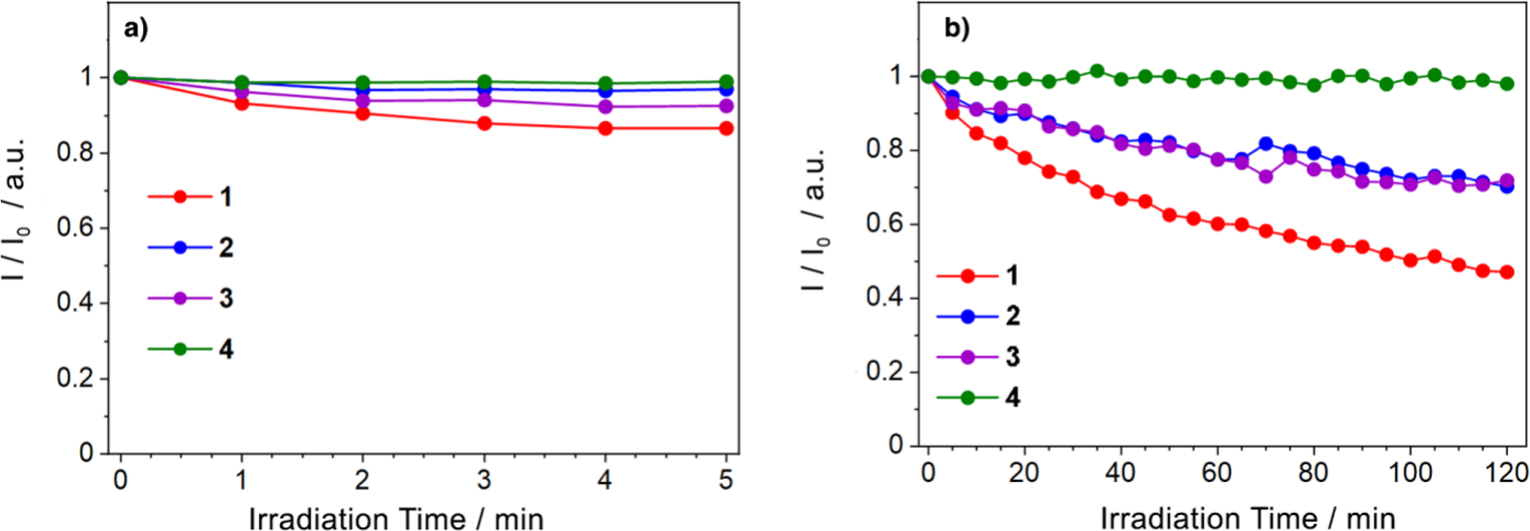
Photostability of complexes **1**–**4** during (a) 5 min and (b) 120 min of continuous irradiation. λ_exc_ = 350 nm.

In luminescent thermometers based on the intensity of a single transition, the photochemical stability of the complex represents a further relevant parameter to be evaluated. Indeed, since Δ corresponds to the integrated intensity of the transition (i.e., the Eu^3+^^5^D_0_ → ^7^F_2_ transition in our case), all phenomena that can modulate the intensity, excluding temperature, are potential sources of uncertainty or error. Photodegradation involves the organic part of the metal complex that is unstable toward oxidation in its excited state in the presence of dioxygen,^64^ thus influencing the service life of the luminescent thermometer. We tested the photostability of our samples in air by monitoring the intensity of the europium ^5^D_0_ → ^7^F_2_ transition (*I*) in two different time lapses of continuous irradiation (Figure 8 and Figure S8): (i) 5 min, which corresponds to the total sample irradiation time during the three cooling/heating cycles (collection of ca. 50 emission spectra), and (ii) 2 h, to simulate harsh working conditions for the luminescent thermometer.

As is visible from Figure 8a, we do not observe for any of the samples a relevant intensity decrease upon irradiation during the first 5 min (Δ*I* < 10%), indicating their photostability in this time lapse. Looking instead at the overall temporal stability of the complexes (Figure 8b), we clearly observe its dependence on the nature of the β-diketonato ligands. In particular, compound **4** does not show relevant photodegradation during the exposition time due to the high stability of C–F bonds.^65^ At variance, complexes **2** and **3** show analogous photodegradation curves and a good photostability, independent of the number of −CF_3_ groups. The intensity of the ^5^D_0_ → ^7^F_2_ transition in these complexes decreased by about 30% of the initial value after 2 h of continuous irradiation. Finally, compound **1** shows the highest fading tendency, keeping only 50% of the initial emission intensity. In this case, the poorer photostability can be attributed to the thienyl ring, which can be easily oxidized under these conditions, as also reported in the literature.^66^

The effect of *N*-oxide ligands does not clearly show up in complexes bearing tta and hfac because it is overshadowed by the photodecomposition paths involving β-diketonato groups. Instead, it can be appreciated only in the **2**–**2b** and **3**–**3b** pairs (Figure S7) where bipyMO derivatives show a lower photobleaching stability in comparison to pyrzMO analogues. It is likely that the absorption properties of the two *N*-oxides play an active role in determining this behavior, the absorption spectrum of bipyMO being red-shifted with respect to those of pyrzMO (Figure S9).

## Conclusions

We studied the correlations between temperature-dependent emission and chemical composition in a family of homodinuclear Eu^3+^ complexes of the general formula [Eu_2_(β-diketonato)_6_(pyrzMO)_*x*_] and [Eu_2_(β-diketonato)_6_(bipyMO)_*x*_] (*x* = 2 for β-diketonato = dbm, bta, and tta; and *x* = 3 for β-diketonato = hfac). The large difference in affinity between nitrogen and oxygen donors in *N*-oxides^41,43^ dictates the reaction outcome for the lanthanides. Irrespective of the employed β-diketonate, *N*-oxide ligands adopted the μ-O bridging coordination mode. Conversely, the number of *N*-oxide molecules coordinated to Eu^3+^ ions is determined by the β-diketonate. These coordination differences for Eu^3+^ ions are closely related to the spectroscopic (absorption and emission) and thermometric properties. With regard to room-temperature photoluminescence, we observed a strong relationship between the complex composition and emission behavior. The values of absolute PLQY and sensitization efficiency η are significantly higher, up to 55% and 96%, respectively, in bipyMO-containing dinuclear complexes. Moreover, we found that the higher the ligand triplet energy, the lower the absolute quantum yields and the greater the η difference between pyrzMO and bipyMO derivatives. These results indicate that nonradiative deactivation processes are more efficient in pyrzMO-based compounds and suggest that they can play a prominent role in TDE. All of the complexes were characterized by a relevant temperature dependence of their photoluminescence properties in the range 223–373 K and displayed a very good thermometric response with *S*_r_ ≥ 1 over an interval of 40 K up to 130 K depending on both the β-diketonato and *N*-oxide ligands. In general, pyrzMO-based complexes show wider exploitable temperature ranges in comparison to their bipyMO analogues. Compound **4** has the largest applicative temperature range (from 243 to 373 K, 130 K temperature interval) and **3** the highest *S*_r_ maximum value, i.e. 7.4% K^–1^ at 373 K. The thermometric properties were characterized by two nonradiative deactivation channels, back energy transfer (BEnT) from the Eu^3+^^5^D_0_ level to the ligand triplet levels and ligand to metal charge transfer (LMCT). In **1**, **2**, **1b**, and **2b** BEnT and LMCT channels are close in energy and both contribute to the quenching of europium emission. For complexes **3**, **4**, **3b**, and **4b**, the high ligand triplet energy (≥21500 cm^–1^) hampers the BEnT mechanism and the LMCT path is the most effective in deactivating the luminescence signal.

## Experimental Section

### Materials and Instrumentation

Anhydrous toluene was purchased from Merck and used as received. [Ln(dbm)_3_], [Ln(tta)_3_], and [Ln(hfac)_3_] (Ln^3+^ = Eu^3+^, Gd^3+^) were prepared according to the literature;^67^ [Ln(bta)_3_] was prepared similarly bytreating [Ln(bta)_3_(H_2_O)_2_]^68^ (Ln^3+^ = Eu^3+^, Gd^3+^) under reduced pressure at 70 °C over P_4_O_10_. PyrzMO was synthesized according to the literature.^69^ FTIR spectra in the solid phase were recorded with a Perkin-Elmer “Spectrum One” spectrometer, equipped with an ATR accessory. Elemental analysis (C, H, N) was performed at the Dipartimento di Chimica e Chimica Industriale, Università di Pisa (Pisa, Italy). Thermogravimetric analysis (TGA) was performed with a TA Instruments SDT 2960 apparatus with simultaneous TGA/DSC system. We followed the mass loss associated with degradation mechanisms as a function of temperature, together with the heat flow. Scans were recorded at a heating rate of 10 °C/min in a temperature range from 293 to 1063 K. Experiments were performed in air with about 10 mg of a microcrystalline sample previously treated under vacuum at room temperature to remove the toluene molecules occluded in the structure. The onset temperature (*T*_onset_), i.e. the temperature value where the weight loss begins, was defined as the intersection point between the baseline and the tangent to the point of maximum slope of the curve oblique side.

Absorption spectra of powder samples were recorded using a Cary 5000 UV–vis spectrometer equipped with an integrating sphere. The spectra were normalized and plotted as *F*(*R*) vs wavelength. *F*(*R*) is the Kubelka–Munk function.^63^

Room-temperature luminescence spectra of sample powders were recorded with a Horiba JobinYvon Fluorolog-3 spectrofluorimeter. Absolute photoluminescence quantum yields (PLQYs) were calculated from corrected emission spectra obtained by means of an integrating sphere. Estimated errors on PLQY and excited-state lifetimes are ±20% and 10%, respectively. A full description of the employed setup can be found in a previous paper.^41^

Since low-temperature (77 K) emission spectra of Gd complexes **5**–**8** and **5b**–**8b** (Figure S3) do not show a well-resolved vibronic progression, i.e. the 0-phonon band cannot be clearly identified, the triplet energy was estimated from the crossing point of the tangent line on the high-energy side of the spectra and the *x* axis.^70,71^ In the literature, the tangent method and the peak-fitting procedure have been used to evaluate the triplet energy in β-diketonato ligands, providing results differing by ca. 3%; therefore we give accordingly an uncertainty of ±3% on these values.^23,72^

Temperature-dependence experiments (223–373 K) were carried using a Horiba T64000 triple spectrometer and a Linkam THMS600 heating/freezing microscope stage having a temperature stability of <0.1 K over a 83–873 K temperature range. A full description of the employed setup has been previously reported.^63^ Mott–Seitz fitting was performed with MATLAB; the curves with *R*^2^ ≥ 0.991 were considered as best-fit curves.

Photostability experiments were conducted in air on solid samples gently pressed on KBr pellets irradiated at 350 nm for 5 min and 2 h alternatively. The integrated intensity of the europium ^5^D_0_ → ^7^F_2_ transition was recorded every 1 and 5 min, respectively. The power of the excitation beam was 60 μW/cm^2^.

### Synthesis of [Eu_2_(tta)_6_(pyrzMO)_2_]·C_7_H_8_ (**1**·C_7_H_8_)

To a solution of [Eu(tta)_3_] (0.208 g, 0.26 mmol) in anhydrous toluene (20 mL) was added pyrzMO (0.025 g, 0.26 mmol). The yellow solution was stirred overnight at room temperature and then concentrated under reduced pressure until precipitation of a yellowish solid, which was filtered and dried *in vacuo* for 5 h (0.15 g, 60.2% yield as [Eu_2_(tta)_6_(pyrzMO)_2_]·C_7_H_8_. El. Anal. Calcd for [Eu_2_(tta)_6_(pyrzMO)_2_]·C_7_H_8_, C_63_H_40_Eu_2_F_18_N_4_O_14_S_6_: C, 39.5; H, 2.1; N, 2.9. Found: C, 39.6; H, 1.8; N, 3.1. IR-ATR (range 1700–700 cm^–1^): 1596s, 1540s, 1507m, 1471m, 1430w, 1411s, 1359m, 1303s, 1061m, 1013w, 934m, 855m, 832w, 784s, 766w, 746w, 716s. Well-shaped single crystals were obtained through diffusion of pentane vapors in a toluene solution of the product. X-ray diffraction studies showed the composition [Eu_2_(tta)_6_(pyrzMO)_2_]·5C_7_H_8_.

The Gd^3+^ derivative [Gd_2_(tta)_6_(pyrzMO)_2_] (**5**) has been obtained following a similar procedure: [Gd(tta)_3_] (0.504 g, 0.61 mmol), pyrzMO (0.059 g, 0.61 mmol). **5**: 0.376 g, 59.8% yield as [Gd_2_(tta)_6_(pyrzMO)_2_]. Anal. Calcd for [Gd_2_(tta)_6_(pyrzMO)_2_], C_56_H_32_F_18_Gd_2_N_4_O_14_S_6_: C, 39.3; H, 2.1; N, 2.9. Found: C, 39.0; H, 1.7; N, 3.1. IR-ATR (range 1700–700 cm^–1^): 1596s, 1540s, 1507m, 1471m, 1430w, 1411s, 1359m, 1303s, 1061m, 1013w, 934m, 855m, 832w, 784s, 766w, 746w, 716s.

### Synthesis of [Eu_***2***_(dbm)_6_(pyrzMO)_2_] (**2**)

A suspension of [Eu(dbm)_3_] (0,192 g, 0.23 mmol) and pyrzMO (0.024 g, 0.235 mmol) in anhydrous toluene (20 mL) was heated at 60 °C for 1 h. The obtained yellow solution was slowly cooled to −20 °C. The crystalline solid that precipitated out was decanted and dried *in vacuo* for 3 h (0.119 g, yield 56.4% as [Eu_2_(dbm)_6_(pyrzMO)_2_]). Anal. Calcd for [Eu_2_(dbm)_6_(pyrzMO)_2_], C_98_H_74_Eu_2_N_4_O_14_: C, 64.1; H, 4.1; N, 3.0. Found: C, 64.4; H, 4.0; N, 2.8. IR-ATR (range 1700–700 cm^–1^): 1592s, 1550s, 1515s, 1473s, 1457s, 1414s, sh, 1305m, 1261w, 1247w, 1218m, 1176w, 1156w, 1068m, 1021m, 1010m, 939w, 850m, 829m, 781w, 757s, 725s. Crystals suitable for single-crystal X-ray diffraction studies were obtained through the diffusion of pentane vapors in a toluene solution of the product.

### [Gd_2_(dbm)_6_(pyrzMO)_2_] (**6**)

[Gd(dbm)_3_] (0.373 g, 0.45 mmol), pyrzMO (0.043 g, 0.45 mmol). **6**: 0.205 g, 50.4% yield as [Gd_2_(dbm)_6_(pyrzMO)_2_]. Anal. Calcd for [Gd_2_(dbm)_6_(pyrzMO)_2_], C_98_H_74_Gd_2_N_4_O_14_,: C, 63.8; H, 4.0; N, 3.0. Found: C, 63.6; H, 3.9; N, 2.9 IR-ATR (range 1700–700 cm^–1^): 1592s, 1550s, 1515s, 1473s, 1457s, 1414s, sh, 1305m, 1261w, 1247w, 1218m, 1176w, 1156w, 1068m, 1021m, 1010m, 939w, 850m, 829m, 781w, 757s, 725s.

### Synthesis of [Eu_2_(bta)_6_(pyrzMO)_2_] (**3**)

To a solution of [Eu(bta)_3_] (0.463 g, 0.58 mmol) in anhydrous toluene (30 mL) was added pyrzMO (0.056 g, 0.58 mmol). The pale yellow solution was stirred at room temperature for 4 h, concentrated under reduced pressure, and then cooled to −20 °C. The colorless solid that formed was filtered and dried *in vacuo* for 7 h (0.32 g, yield 62.0% as [Eu_2_(bta)_6_(pyrzMO)_2_]). Anal. Calcd for [Eu_2_(bta)_6_(pyrzMO)_2_], C_68_H_44_Eu_2_F_18_N_4_O_14_: C, 45.7; H, 2.5; N, 3.1. Found: C, 45.7; H, 2.5; N, 3.5. IR-ATR (range 1700–700 cm^–1^): 1632m, 1608s, 1574m, 1532m, 1473m, 1431w, 1319m, 1292s, 1242m, 1189m, 1130s, 1077m, 1025w, 1011w, 945w, 848m, 833m, 810w, 767m, 716m, 700m. Crystals suitable for single-crystal X-ray diffraction studies were obtained through diffusion of pentane vapors in a toluene solution of the product. When the reaction was repeated using an [Eu(bta)_3_]/pyrzMO molar ratio of 2/3, compound **3** was still obtained.

### [Gd_***2***_(bta)_6_(pyrzMO)_2_] (**7**)

[Gd(bta)_3_] (0.498 g, 0.62 mmol), pyrzMO (0.060 g, 0.62 mmol). **7**: 0.377 g, 67.7% yield as [Gd_2_(bta)_6_(pyrzMO)_2_]. Anal. Calcd for [Gd_2_(bta)_6_(pyrzMO)_2_], C_68_H_44_F_18_Gd_2_N_4_O_14_: C, 45.4; H, 2.5; N, 3.1. Found: C, 45.3; H, 2.3; N, 3.0. IR-ATR (range 1700–700 cm^–1^): 1632m, 1608s, 1574m, 1532m, 1473m, 1431w, 1319m, 1292s, 1242m, 1189m, 1130s, 1077m, 1025w, 1011w, 945w, 848m, 833m, 810w, 767m, 716m, 700m.

### Synthesis of [Eu_2_(hfac)_6_(pyrzMO)_3_] (**4**)

To a suspension of [Eu(hfac)_3_] (0.766 g; 0.99 mmol) in toluene (30 mL) was added pyrzMO (0.143 g; 1.49 mmol). The colorless mixture was refluxed for 1 h. The resulting pale yellow solution was cooled to room temperature, concentrated under reduced pressure, and finally cooled to −20 °C. Precipitation of a colorless crystalline solid occurred. The suspension was filtered, and the solid was dried *in vacuo* for 4 h (0.52 g; 57.3% yield as [Eu_2_(hfac)_6_(pyrzMO)_3_]). Anal. Calcd for [Eu_2_(hfac)_6_(pyrzMO)_3_], C_42_H_18_Eu_2_F_36_N_6_O_15_: C, 27.5; H, 1.0; N, 4.6. Found: C, 27.5; H, 1.4; N, 4.4. IR-ATR (range 1700–700 cm^–1^): 1648m, 1577w, 1533w, 1495m, 1473m, 1435m, 1253m, 1202m, 1138s, 1098m, 1012m, 848m, 801m, 734w,716w. Recrystallization from toluene at −20 °C afforded crystals suitable for X-ray diffraction studies showing the composition [Eu_2_(hfac)_6_(pyrzMO)_3_]·C_7_H_8_. The crystals were treated under an atmosphere saturated with toluene to avoid collapse due to the loss of the crystallization solvent.

### [Gd_2_(hfac)_6_(pyrzMO)_3_] (**8**)

[Gd(hfac)_3_] (0.498 g, 0.64 mmol), pyrzMO (0.093 g, 0.96 mmol). **8**: 0.356 g, 60.3% yield as [Gd_2_(hfac)_6_(pyrzMO)_3_]. Anal. Calcd for [Gd_2_(hfac)_6_(pyrzMO)_3_], C_42_H_18_F_36_Gd_2_N_6_O_15_: C, 27.3; H, 1.0; N, 4.6. Found: C, 27.0; H, 1.1; N, 4.5. IR-ATR (range 1700–700 cm^–1^): 1648m, 1577w, 1533w, 1495m, 1473m, 1435m, 1253m, 1202m, 1138s, 1098m, 1012m, 848m, 801m, 734w, 716w.

### X-ray Diffraction Study

Crystals of **2** and **3** and were glued at the end of glass fibers, and those of **1, 3b**, and **4** were closed in Lindeman capillaries under a toluene-saturated nitrogen atmosphere. Diffractions were studied at room temperature by means of a Bruker SMART Breeze CCD diffractometer equipped with graphite-monochromated Mo Kα radiation (λ = 0.71073 Å). The crystal data are given in Table 4. Intensity data collections were carried out for all samples within a maximum 2θ of about 52°, because the diffractions beyond this limit were very weak. All of the structure solutions were found using the automated direct methods contained in the SHELXS-97 program.^63^ Crystal structures of **1**, **3b**, and **4** contained, in addition to the dinuclear molecule, also toluene solvate. In the case of **3b**, the solvent molecules were so disordered that it was impossible to localize the exact position, and their contribution was subtracted from the electron density map by the SQUEEZE procedure.^64^ In the crystal structure of **1** there were five toluene molecules for each dinuclear metal complex, one of them being placed on an inversion center. The disorder present in a CF_3_ group of the crystal structure of **3** made it necessary to consider it as distributed in two limit positions. The reliability factors obtained in the final refinement cycles are given in Table 4. Supplementary crystallographic data for this paper have been deposited with The Cambridge Crystallographic Data Centre and can be obtained free of charge from it. The deposition numbers for each compound are given in Table 4.

**Table 4:** Crystal Data and Refinement Summaries for **1**–**4**

	**1**·5(toluene)	**2**	**3**	**4**·(toluene)
CCDC no.	2008931	2008932	2008933	2008935
empirical formula	C_91_H_72_Eu_2_F_18_N_4_O_14_S_6_	C_98_H_74_Eu_2_N_4_O_14_	C_68_H_44_Eu_2_F_18_N_4_O_14_	C_49_H_26_Eu_2_F_36_N_6_O_15_
formula wt	2283.80	1835.58	1786.99	1926.68
cryst syst	monoclinic	monoclinic	monoclinic	monoclinic
space group	*P*2_1_/*c*	*P*2_1_/*n*	*P*2_1_/*n*	*P*2_1_/*n*
*a* (Å)	12.69(3)	14.2487(7)	21.1218(10)	13.474(3)
*b* (Å)	24.14(6)	19.861(1)	15.6107(7)	20.621(4)
*c* (Å)	17.14(4)	16.1236(8)	22.8947(10)	25.124(5)
β (deg)	102.76(4)	111.021(1)	106.864(2)	97.30(3)
Volume (Å^3^)	5121(22)	4259.2(4)	7224.3(6)	6924(3)
*Z*	2	2	4	4
ρ_calc_ (g cm^–3^)	1.481	1.431	1.643	1.848
μ (mm^–1^)	1.427	1.526	1.831	1.955
*F*(000)	2284	1856	3520	3728
no. of data/restraints/params	9848/126/595	10560/0/532	14508/280/993	14310/755/923
goodness of fit on *F*^2^	1.185	1.126	1.173	1.062
final R1 (*I* ≥ 2σ(*I*))	0.0861	0.0359	0.0699	0.0900
final wR2 (*I* ≥ 2σ(*I*))	0.1882	0.0755	0.1045	0.2602
final R1 (all data)	0.1234	0.0473	0.1192	0.1109
final wR2 (all data)	0.2102	0.0807	0.1210	0.2844

## Supporting Information Available

Syntheses of bipyMO-based compounds (**3b**, **5b**–**8b**), crystallographic data for **3b**, thermogravimetric analyses, Gd^3+^ derivative PL measurements, and additional thermometric studies (PDF)

## Accession Codes

CCDC 2008931–2008935 contain the supplementary crystallographic data for this paper. These data can be obtained free of charge via www.ccdc.cam.ac.uk/data_request/cif, or by emailing data_request@ccdc.cam.ac.uk, or by contacting The Cambridge Crystallographic Data Centre, 12 Union Road, Cambridge CB2 1EZ, UK; fax: +44 1223 336033.
