# Supplementary material for: Composition–Thermometric Properties Correlations in Homodinuclear Eu3+ Luminescent Complexes
Source: Inorg Chem. 2020 Dec 10;59(24):18156–67. doi: 10.1021/acs.inorgchem.0c02611 (PMC8016189; doi:10.1021/acs.inorgchem.0c02611)
Supplement: Supplementary file 1 — ic0c02611_si_001.pdf [file ic0c02611_si_001.pdf]

Inorganic Chemistry

Revised Manuscript ID ic-2020-02611r.R1

Supporting information

Composition-thermometric properties correlations in homodinuclear Eu^{3+} luminescent complexes

Luca Bellucci[†§], Gregorio Bottaro[†§*], Luca Labella[‡†*], Valerio Causin[§], Fabio Marchetti[‡], Simona Samaritani[‡], Daniela Belli Dell'Amico[‡], Lidia Armelao[§†].

[†]*CNR ICMATE and INSTM, Dipartimento di Scienze Chimiche, Università di Padova, via Marzolo 1,I-35131*

[‡]*Dipartimento di Chimica e Chimica Industriale and CIRCC, Università di Pisa, via Giuseppe Moruzzi 13, I-56124*

[§]*Dipartimento di Scienze Chimiche, Università di Padova, via Marzolo 1,I-35131*

Corresponding authors' email address:

gregorio.bottaro@cnr.it

luca.labella@unipi.it

Table of contents Supporting Information

Experimental section for bipyMO-based dinuclear complexes

- *Synthesis of [Eu_2(bta)$_6$(bipyMO)$_2$]* (3b)
- *Synthesis of [Gd_2(tta)$_6$(bipyMO)$_2$]* (5b)
- *Synthesis of [Gd_2(dbm)$_6$(bipyMO)$_2$]* (6b)
- *Synthesis of [Gd_2(bta)$_6$(bipyMO)$_2$]* (7b)
- *Synthesis of [Gd_2(hfac)$_6$(bipyMO)$_3$]* (8b)

List of tables

- **Table S1:** Crystal data and refinement summaries for compound **3b.**
- **Table S2:** Coordination polyhedra of compounds **1**, **2**, **3**, and **4.**

List of figures

- **Figure S1:** Molecular structure of compound **3b**; only the most populated positions of disordered CF_3 groups have been represented.

- **Figure S2: a)** Photoluminescence excitation spectra (PLE, λ_{em}= 611 nm) and **b)** emission spectra of compound **3b** ($\lambda_{exc=}$ 350 nm).
- **Figure S3:** Low temperature (77K) emission spectra of **5-8** and **5b-8b** Gd^{3+}-complexes. The spectra, because of their broadness and of the lack of a well-resolved vibronic progression, do not allow a precise determination of the 0-phonon line. For this reason we estimated the triplet energy values by tangent line on the high energy side of the spectra. The obtained values are in agreement with the literature values for employed β-diketonato ligands. The small differences in **5-5b**, **6-6b**, **7-7b**, **8-8b** are within the experimental errors (3 %).
- **Figure S4:** TGA curves of compounds **a) 1**, **b) 2**, **c) 3**, **d) 4**, **e) 1b**, f) **2b**, **g) 3b**, and **h) 4b**.
- **Figure S5:** Temperature-dependent emission spectra in the 223 - 373 K temperature range of compounds **a) 1, b) 2**, **c) 3**, **d) 4**, **e) 1b**, **f) 2b**, **g) 3b** and **h) 4b**.
- **Figure S6:** MS fitting curves of compounds **a) 1**, **b) 2**, **c) 3**, **d) 4**, **e) 1b**, **f) 2b**, **g) 3b**, **h) 4b.**
- **Figure S7:** Overlap of photoluminescence excitation (PLE) and the diffuse reflectance (DR) spectra of compounds **a) 1b**, **b) 2b**, **c) 3b**, and **d) 4b**. The label "0-0" refers to the energy of Eu^{3+} $^5D_0 \rightarrow {}^7F_0$ transition, *T* is the energy of the β-diketonato triplet level, while ΔE_1 is the activation energy found with the MS equation.
- **Figure S8:** Comparison of the complexes photostability: **a) 1** vs **1b**, **b) 2** vs **2b**, **c) 3** vs **3b**, and **d) 4** vs **4b** during 2 h. λ_{exc}= 350 nm.
- **Figure S9:** Absorption spectra of bipyMO and pyrzMO in toluene. The vertical green line highlights the wavelength used in the photostability studies.

Experimental section for bipyMO-based dinuclear complexes

BipyMO was synthesized according to the literature.[1] The complexes $[Eu_2(tta)_6(bipyMO)_2]$ (**1b**), $[Eu_2(dbm)_6(bipyMO)_2]$ (**2b**), and $[Eu_2(hfac)_3(bipyMO)_3]$ (**4b**) were synthesized following the literature procedure.[2]

Synthesis of [Eu₂(bta)₆(bipyMO)₂] **(3b).** To a solution of $[Eu(bta)_3]$ (0.357 g, 0.45 mmol) in anhydrous toluene (25 mL) bipyMO (0.087 g, 0. 50 mmol) was added. The pale-yellow solution was refluxed for 2 h, cooled at room temperature, concentrated under reduced pressure, and then cooled to -20 °C. The colorless precipitate was filtered and dried in vacuo for 5 h. (0.31 g, yield 71.0 % as $[Eu_2(bta)_6(bipyMO)_2]$). El. Anal. Calcd for $[Eu_2(bta)_6(bipyMO)_2]$, $C_{80}H_{52}Eu_2F_{18}N_4O_{14}$, %: Teor. C, 49.5; H, 2.7; N, 2.9. Found: C, 49.3; H, 2.6; N, 2.9. IR-ATR (range: 1700-700 cm^{-1}): 1636m, 1611s, 1575m, 1537m, 1479m, 1319m, 1289s, 1239m, 1179m, 1126s, 1074m, 1024w, 944w, 847w, 816m, 764s, 734m, 716m. Crystal suitable to single crystal X-Ray diffraction studies were obtained through diffusion of pentane vapours in a toluene solution of the product.

Synthesis of $[Gd_2(tta)_6(bipyMO)_2]$ **(5b).** To a solution of $[Gd(tta)_3]$ (0.435 g, 0.53 mmol) in anhydrous toluene (10 mL) bipyMO (0.091 g, 0. 53 mmol) was added. The yellow solution was refluxed for 2 h, then concentrated under reduced pressure and subsequently cooled at -20 °C. The resulting yellowish solid was filtered and dried in vacuo for 5 h (0.281 g, 53.5 % yield as $[Gd_2(tta)_6(bipyMO)_2]$). El. Anal. Calcd for $[Gd_2(bta)_6(bipyMO)_2]$, $C_{68}H_{40}F_{18}Gd_2N_4O_{14}S_6$, %: Teor. C, 41.1; H, 2.0; N, 2.8. Found: C, 41.0; H, 1.9; N, 2.6. IR-ATR (range: 1700-700 cm^{-1}): 1620m, 1596s, 1537s, 1505m, 1473m, 1412m, 1351w, 1304s, 1245m, 1229m, 1179s, 1131s, 1082w, 1061 m, 1032w, 1017w, 991w, 949w, 935w, 859w, 847w, 813m, 785s, 767w, 749w, 714s.

Synthesis of $[Gd_2(dbm)_6(bipyMO)_2]$ **(6b).** To a solution of $[Gd(dbm)_3]$ (0.380 g, 0.46 mmol) in anhydrous toluene (10 mL) bipyMO (0.079 g, 0. 46 mmol) was added. The pale-yellow solution was refluxed for 2 h, concentrated under reduced pressure at room temperature, and then cooled to -20 °C. The obtained yellow precipitate was filtered and dried in vacuo for 4 h. (0.308 g, yield 67.0 % as $[Gd_2(dbm)_6(bipyMO)_2]$). El. Anal. Calcd for $[Gd_2(dbm)_6(bipyMO)_2]$, $C_{110}H_{82}Gd_2N_4O_{14}$, %: Teor. C, 66.1; H, 4.1; N, 2.8. Found: C, 66.0; H, 4.0; N, 2.8. IR-ATR (range: 1700-700 cm^{-1}): 1594s, 1549s, 1513s, 1477s, 1456s, 1412s, 1391m, 1306m, 1283m, 1217m, 1178w, 1155w, 1067 m, 1022m, 940w, 848w, 811m, 782w, 745m, 719s.

Synthesis of $[Gd_2(bta)_6(bipyMO)_2]$ **(7b).** To a suspension of $[Gd(bta)_3]$ (0.425 g, 0.53 mmol) in anhydrous toluene (10 mL) bipyMO (0.091 g, 0. 53 mmol) was added. The pale-yellow suspension was refluxed for 2 h obtaining a solution which was cooled to room temperature, concentrated under reduced pressure and finally cooled to -20 °C. Precipitation of a colorless solid occurred. The suspension was filtered and the solid was dried in vacuo for 4 h (0.268 g, yield 52.0 % as $[Gd_2(bta)_6(bipyMO)_2]$). El. Anal. Calcd for $[Gd_2(bta)_6(bipyMO)_2]$, $C_{80}H_{52}F_{18}Gd_2N_4O_{14}$, %: Teor. C, 49.3; H, 2.7; N, 2.9. Found: C, 49.1; H, 2.5; N, 2.9. IR-ATR (range: 1700-700 cm^{-1}): 1636m, 1611s, 1575m, 1537m, 1479m, 1319m, 1289s, 1239m, 1179m, 1126s, 1074m, 1024w, 944w, 847w, 816m, 764s, 734m, 716m.

Synthesis of $[Gd_2(hfac)_6(bipyMO)_3]$ **(8b).** To a suspension of $[Gd(hfac)_3]$ (0.272 g, 0.35 mmol) in anhydrous toluene (10 mL) bipyMO (0.091 g, 0. 53 mmol) was added. The colorless suspension was refluxed for 2 h. The obtained solution was cooled at room temperature, concentrated under reduced pressure, and then cooled to -20 °C. The colorless precipitate was filtered and dried in vacuo for 4 h. (0.184 g, yield 50.8 % as $[Gd_2(hfac)_6(bipyMO)_3]$). El. Anal. Calcd for $[Gd_2(hfac)_6(bipyMO)_3]$, $C_{60}H_{30}F_{36}Gd_2N_6O_{15}$, %: Teor. C, 34.8; H, 1.5; N, 4.1. Found: C, 34.6; H, 1.5; N, 3.9. IR-ATR (range: 1700-700 cm^{-1}): 1663m, 1649s, 1601w, 1555m, 1528m, 1505m, 1481m, 1412w, 1348w, 1321w, 1251s, 1196s, 1135s, 1097s, 1040w, 950w, 850m, 814m, 795s, 767 w, 739m, 719w.

Bibliography

(1) Brunner, H.; Störiko, R.; Rominger, F. Novel Chiral Oxazoline Ligands for Potential Charge-Transfer Effects in the Rh(I)-Catalysed Enantioselective Hydrosilylation. *Eur. J. Inorg. Chemsitry* **1998**, 771–781.

(2) Armelao, L.; Belli Dell'Amico, D.; Bellucci, L.; Bottaro, G.; Ciattini, S.; Labella, L.; Manfroni, G.; Marchetti, F.; Mattei, C. A.; Samaritani, S. Homodinuclear Lanthanide Complexes with the Divergent Heterotopic 4,4′-Bipyridine N -Oxide (BipyMO) Ligand. *Eur. J. Inorg. Chem.* **2018**, *40*, 4421–4428.

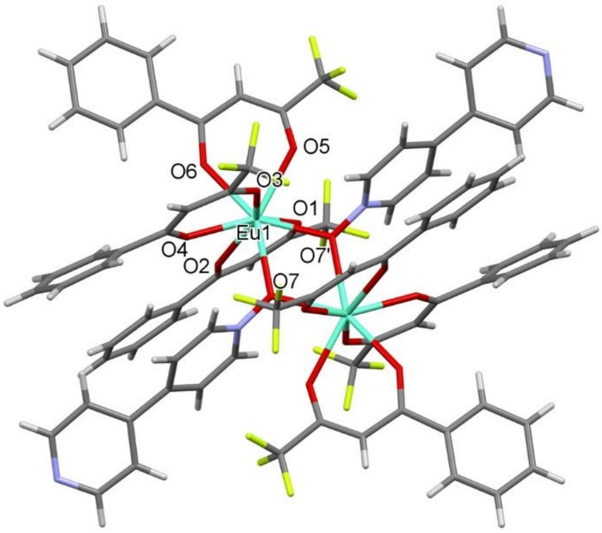

Figure S1: Molecular structure of compound **3b**.

Table S1: Crystal data and refinement summaries for compound **3b**.

Identification code	**3b** [+ toluene]
CCDC number	2008934
Empirical formula	$C_{80}H_{52}Eu_2F_{18}N_4O_{14}$ [+ toluene]
Formula weight	1939.17
Crystal system	Monoclinic
Space group	$P2_1/c$
a [Å]	12.8640(4)
b [Å]	14.3713(4)
c [Å]	23.6334(7)
β [°]	95.9290(10)
Volume [Å^3]	4345.8(2)
Z	2
ρ_{calc} [g cm^{-3}]	1.482
μ [mm^{-1}]	1.529
F(000)	1920
Data/restraints/parameters	8707 / 0 / 532
Goodness-of-fit on F^2	1.042
Final R_1 [$I \geq 2\sigma(I)$]	0.0529
Final wR_2 [$I \geq 2\sigma(I)$]	0.1474
Final R_1 [all data]	0.0691
Final wR_2 [all data]	0.1603

Table S2: Coordination polyhedra of compounds **1**, **2**, **3**, and **4**.

Coordination polyhedron of compound **1**.

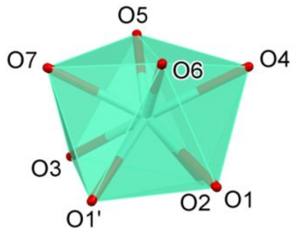

Eu1–O1	2.488(7)
Eu1–O2	2.373(9)
Eu1–O3	2.415(7)
Eu1–O4	2.372(8)
Eu1–O5	2.353(8)
Eu1–O6	2.424(8)
Eu1–O7	2.388(7)
Eu1–O1′	2.505(8)

Coordination polyhedron of compound **2**.

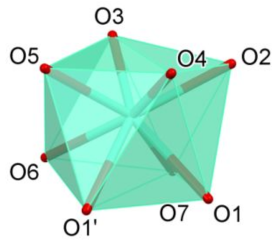

Eu1–O1	2.4744(19)
Eu1–O2	2.364(2)
Eu1–O3	2.334(2)
Eu1–O4	2.396(2)
Eu1–O5	2.3470(19)
Eu1–O6	2.338(2)
Eu1–O7	2.356(2)
Eu1–O1′	2.5403(18)

Coordination polyhedra of compound **3**.

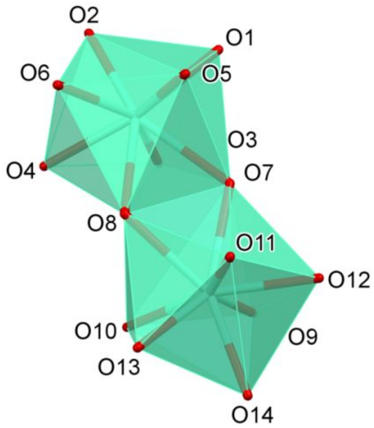

Eu1–O1	2.349(5)
Eu1–O2	2.372(5)
Eu1–O3	2.378(5)
Eu1–O4	2.330(5)
Eu1–O5	2.385(5)
Eu1–O6	2.339(4)
Eu1–O7	2.452(5)
Eu1–O8	2.543(5)
Eu2–O7	2.481(5)
Eu2–O8	2.451(4)
Eu2–O9	2.353(5)
Eu2–O10	2.374(6)
Eu2–O11	2.372(6)
Eu2–O12	2.341(5)
Eu2–O13	2.375(5)
Eu2–O14	2.323(6)

Coordination polyhedra of compound **4**.

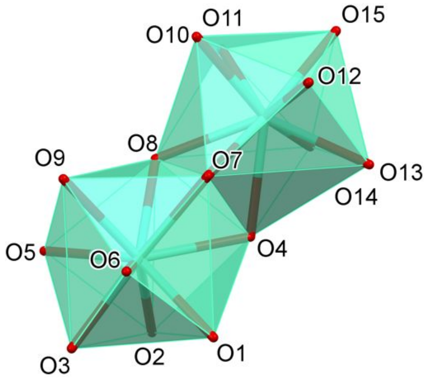

Eu1–O1	2.389(8)
Eu1–O2	2.370(8)
Eu1–O3	2.371(8)
Eu1–O4	2.546(9)
Eu1–O5	2.414(8)
Eu1–O6	2.409(8)
Eu1–O7	2.475(8)
Eu1–O8	2.538(9)
Eu1–O9	2.437(8)
Eu2–O4	2.502(9)
Eu2–O7	2.513(8)
Eu2–O8	2.522(8)
Eu2–O10	2.408(8)
Eu2–O11	2.429(8)
Eu2–O12	2.449(8)
Eu2–O13	2.398(8)
Eu2–O14	2.408(8)
Eu2–O15	2.339(9)

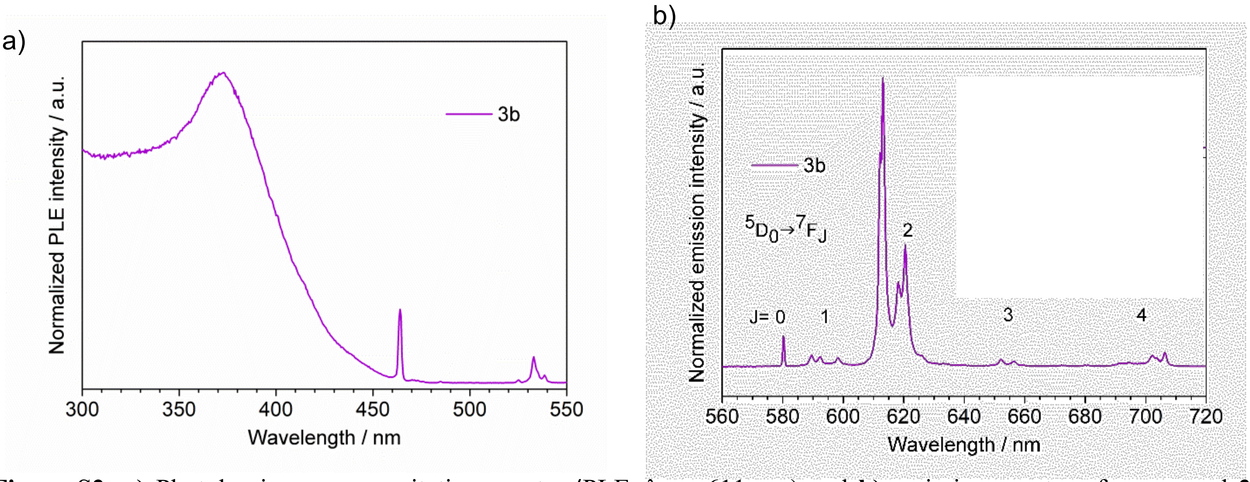

Figure S2: a) Photoluminescence excitation spectra (PLE, λ_{em}= 611 nm) and **b)** emission spectra of compound **3b** ($\lambda_{exc=}$ 350 nm).

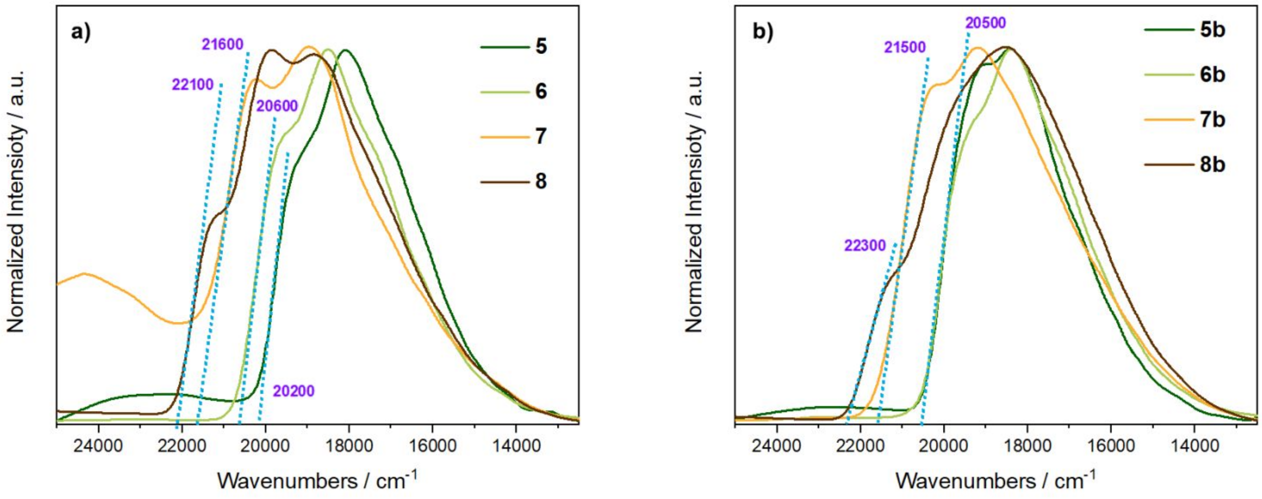

Figure S3: Low temperature (77K) emission spectra of **5-8** and **5b-8b** Gd^{3+}-complexes. The spectra, because of their broadness and of the lack of a well-resolved vibronic progression, do not allow a precise determination of the 0-phonon line. For this reason we estimated the triplet energy values by tangent line on the high energy side of the spectra. The obtained values are in agreement with the literature values for employed β-diketonato ligands. The small differences in **5-5b**, **6-6b**, **7-7b**, **8-8b** are within the experimental errors (3 %).

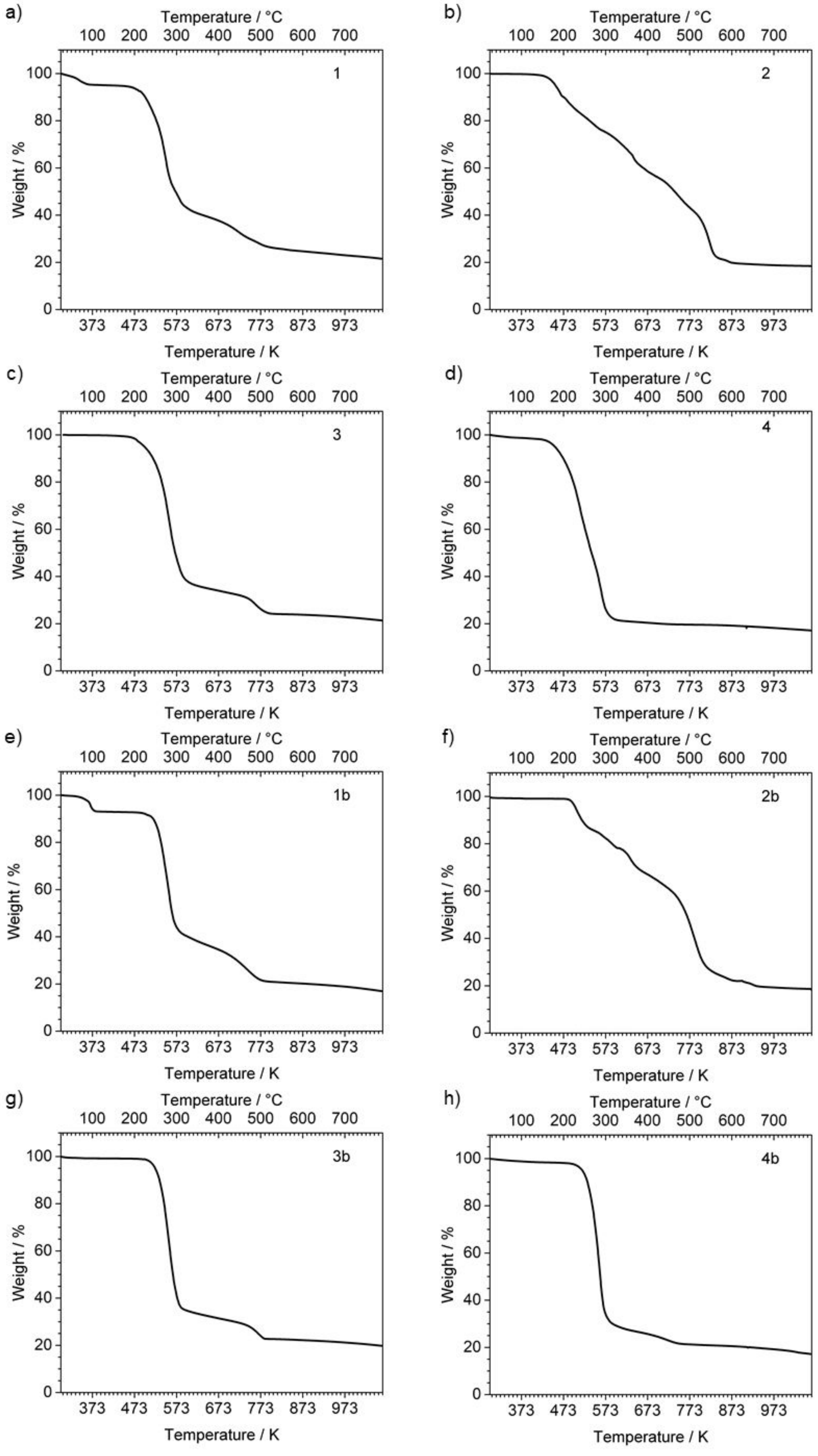

Figure S4: TGA curves of compounds **a) 1**, **b) 2**, **c) 3**, **d) 4**, **e) 1b**, f) **2b**, **g) 3b**, and **h) 4b**.

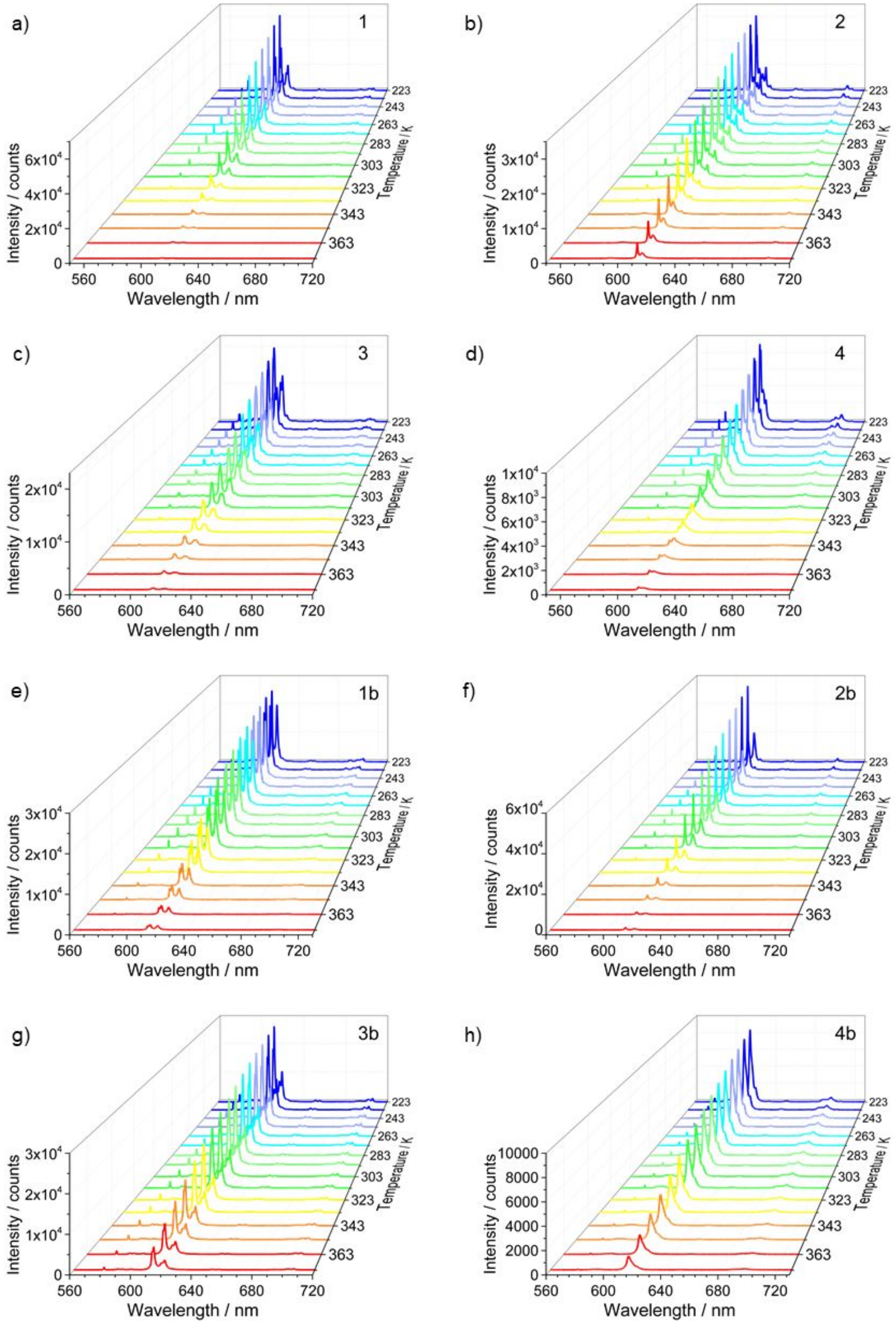

Figure S5: Temperature-dependent emission spectra in the 223 - 373 K temperature range of compounds **a) 1, b) 2**, **c) 3**, **d) 4**, **e) 1b**, **f) 2b**, **g) 3b** and **h) 4b**.

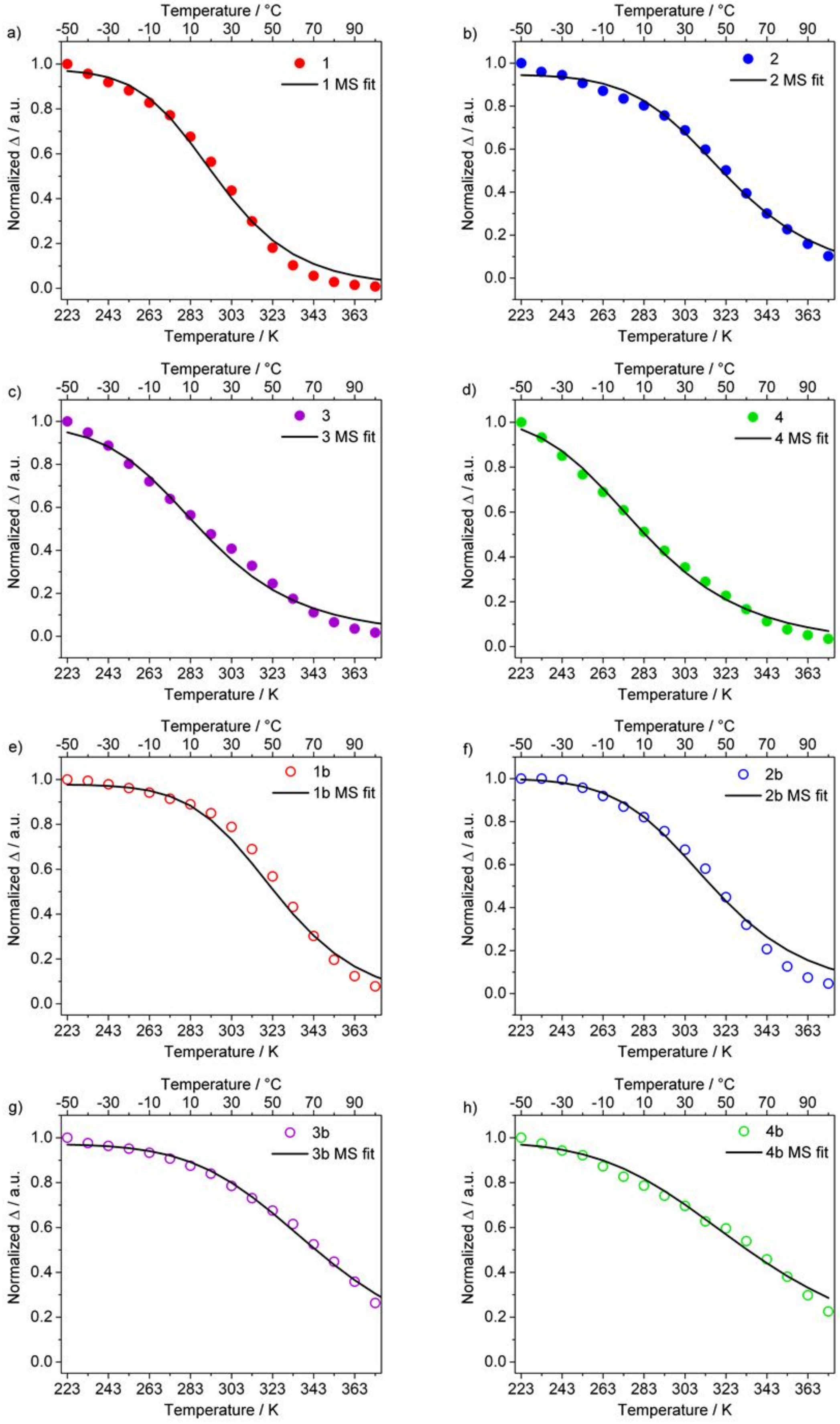

Figure S6: MS fitting curves of compounds **a) 1**, **b) 2**, **c) 3**, **d) 4**, **e) 1b**, **f) 2b**, **g) 3b**, **h) 4b.**

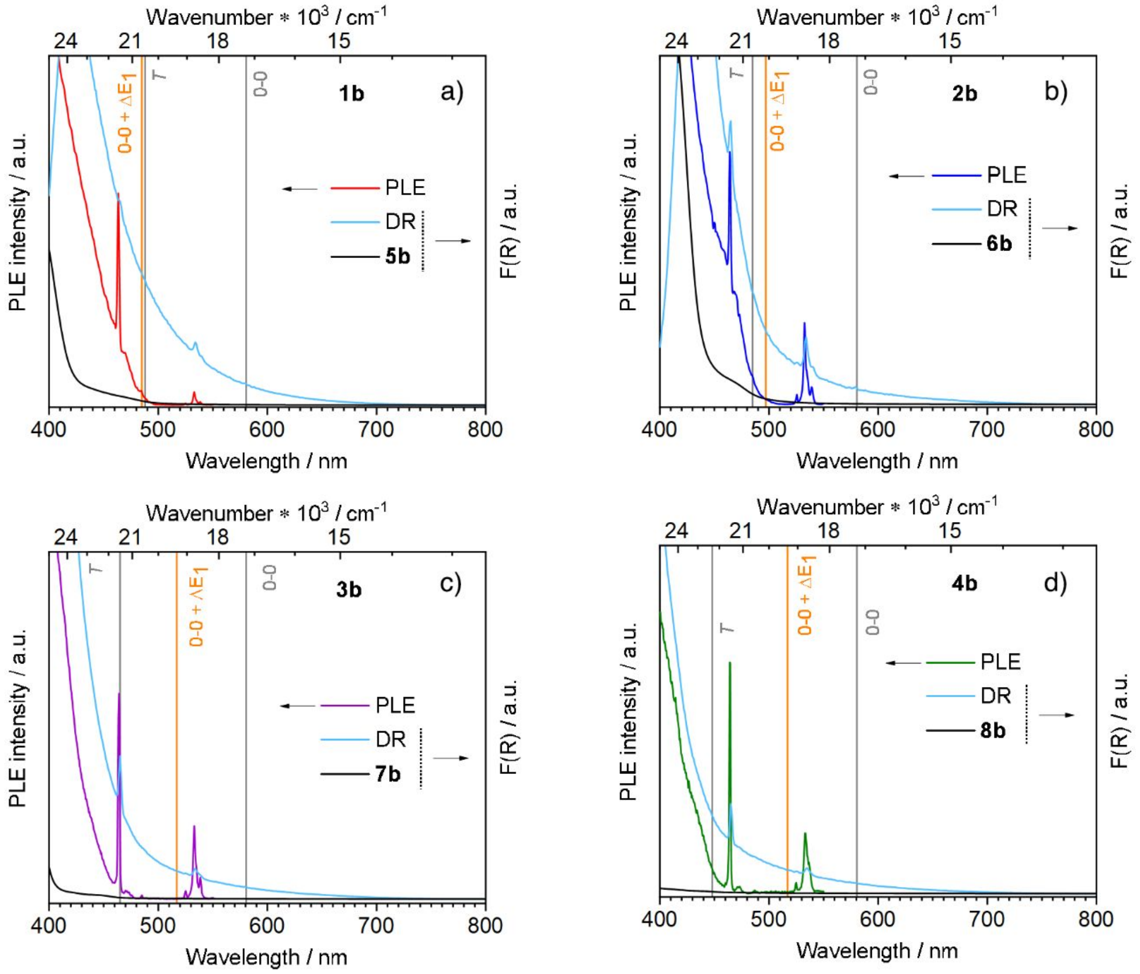

Figure S7: Overlap between the photoluminescence excitation (PLE) and the diffuse reflectance (DR) spectra of compounds **a) 1b**, **b) 2b**, **c) 3b**, and **d) 4b**. To better evidence the presence of LMCT transitions the absorption spectra of Gd-complexes **5b**, **6b**, **7b**, **8b** are reported as well. The label "0-0" refers to the energy of Eu^{3+} $^5D_0 \rightarrow ^7F_0$ transition, *T* is the energy of the β-dike triplet level, while ΔE_1 is the activation energy found with the MS equation.

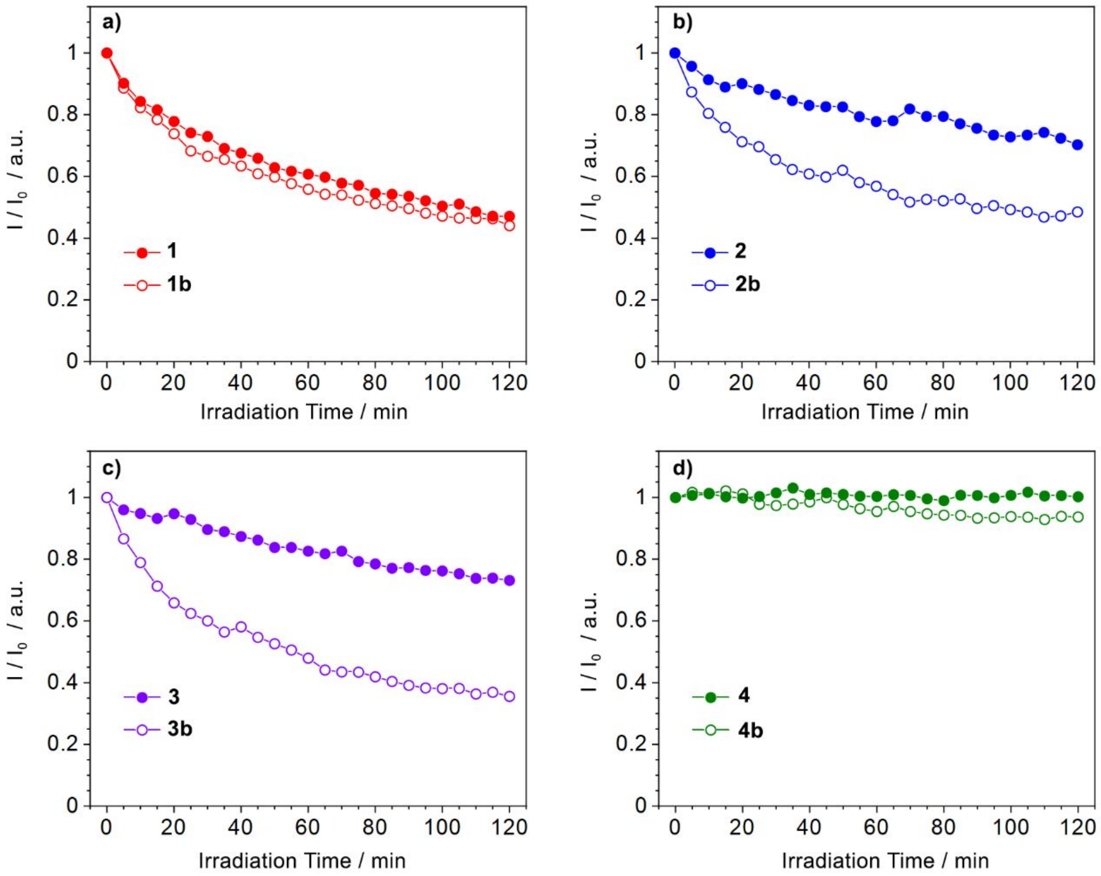

Figure S8: Comparison of the complexes photostability: **a) 1** vs **1b**, **b) 2** vs **2b**, **c) 3** vs **3b**, and **d) 4** vs **4b** during 120 min of continuous irradiation at λ_{exc}= 350 nm.

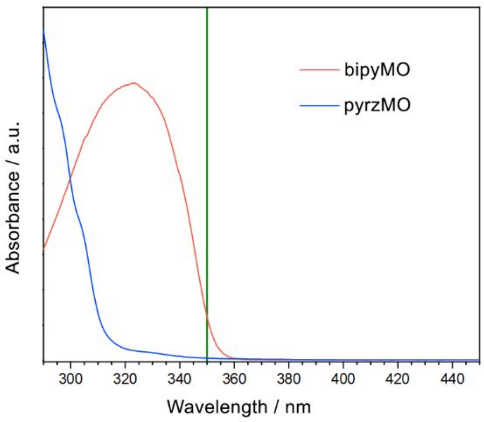

Figure S9: Absorption spectra of bipyMO and pyrzMO in toluene. The vertical green line highlights the wavelength used in the photostability studies.
